# Glucose‐induced LINC01419 reprograms the glycolytic pathway by recruiting YBX1 to enhance PDK1 mRNA stability in hepatocellular carcinoma

**DOI:** 10.1002/ctm2.70122

**Published:** 2024-12-03

**Authors:** Yanfang Liu, Junjiao Song, Qili Shi, Bing Chen, Wenying Qiu, Yizhe Liu, Shenglin Huang, Xianghuo He

**Affiliations:** ^1^ Department of Oncology Shanghai Medical College Fudan University Shanghai Cancer Center and Institutes of Biomedical Sciences Fudan University Shanghai China; ^2^ Key Laboratory of Breast Cancer in Shanghai Fudan University Shanghai Cancer Center Fudan University Shanghai China; ^3^ Collaborative Innovation Center for Cancer Personalized Medicine Nanjing University Nanjing China

**Keywords:** glycolytic pathway, hepatocellular carcinoma, LINC01419, mRNA stability, PDK1

## Abstract

Metabolic reprogramming provides the necessary energy for the development of malignant tumours and is emerging as a novel tumour treatment strategy. However, the widespread expression of metabolic enzymes in diverse cell types makes the development of specific drugs that target cancer cells without affecting normal cellular functions challenging. Accumulating evidence has demonstrated the essential roles of long non‐coding RNAs (lncRNAs) in the regulatory network associated with glucose metabolism in tumour cells. The mechanism and therapeutic potential of cancer‐specific lncRNAs in modulating tumour glucose metabolism warrant in‐depth exploration. Here we revealed that glucose‐induced LINC01419 promoted the growth and metastasis of HCC cells by driving metabolic reprogramming. Mechanistically, LINC01419 directly interacted with Y‐box binding protein 1 (YBX1) in the cytoplasm and facilitated its binding to PDK1 mRNA, thus enhancing PDK1 mRNA stability and increasing lactate production. Furthermore, YY1 contributed to the transcriptional activation of LINC01419 in HCC under high‐glucose conditions. Notably, administration of an N‐acetylgalactosamine (GalNAc)‐conjugated siRNA specifically targeting LINC01419 markedly retarded the growth of orthotopic xenograft tumours. These findings provide evidence for an unprecedented regulatory mechanism of LINC01419 involving metabolic reprogramming in human cancer. The newly identified LINC01419/YBX1‐PDK1 axis may represent a promising therapeutic target for HCC. Moreover, GalNAc‐siLINC01419 holds significant potential for clinical application.

**Key points:**

- This study highlights the considerable regulatory role of LINC01419 in the metabolism of HCC.
- The newly identified LINC01419/YBX1‐PDK1 axis constitutes a valuable target.
- Hepatic‐specific delivery of GalNAc‐siLINC01419 presents a promising therapeutic strategy for HCC.

## Graphical abstract

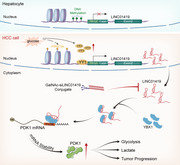

## 1 INTRODUCTION

Metabolic abnormalities are important characteristics of human cancers, and tumour cells undergo various metabolic reprogramming processes to support malignant growth and metastasis.^1^, ^2^, ^3^ Hepatocellular carcinoma (HCC) is a highly heterogeneous malignancy associated with a poor prognosis,^4^, ^5^ and the available therapeutic strategies have clear limitations. Therefore, the development of innovative and effective strategies for HCC treatment is urgently needed. Metabolic reprogramming is crucial in the occurrence and progression of HCC, with significant and comprehensive reprogramming of glucose metabolism commonly observed in HCC.^6^ The classic metabolic reprogramming phenomenon observed in tumours is aerobic glycolysis, a phenomenon recognized as the Warburg effect.^7^ Given the pivotal role of glycolysis in tumour growth and metastasis, numerous inhibitors targeting glycolytic enzymes have been developed for tumour therapy.^8^ Nonetheless, as glycolytic enzymes are expressed in a wide range of tissues, their inhibitors may interfere with glucose metabolism in normal tissues.^9^ For example, HK2 inhibitors can lead to the degradation of the widely expressed enzyme HK1, which shares a similar structure, thereby impacting metabolism in normal tissue.^10^, ^11^ Thus, the development of innovative approaches to modulate metabolic abnormalities in tumour cells holds great promise for tumour treatment. Pyruvate dehydrogenase kinase 1 (PDK1) is an enzyme that exerts a pivotal influence on metabolic reprogramming by inhibiting the activity of pyruvate dehydrogenase (PDH), and promoting the conversion of pyruvate to lactate, driving the Warburg effect by inhibiting oxidative metabolism and promoting glycolysis.^12^ Elevated levels of PDK1 expression have been implicated in tumour progression, metastasis, and the development of resistance to chemotherapy.^13^, ^14^, ^15^, ^16^ PDK1 is instrumental in facilitating the efficient liver metastasis of breast cancer by mediating metabolic adaptations to a new microenvironment.^14^ PGC1α inhibits the Warburg effect by downregulating the expression of PDK1 through the WNT/β‐catenin pathway, thereby suppressing HCC metastasis.^17^ Inhibition of PDK1 presents an attractive strategy for anticancer therapy, and the potential anticancer effects of several PDK1 inhibitors, such as dichloroacetate (DCA), have been investigated in preclinical and clinical studies.^18^, ^19^ However, the regulation of PDK1 expression and the development of targeted therapies for HCC require further investigation.

Long non‐coding RNAs (lncRNAs) are ubiquitous but exhibit tissue‐specific and disease‐specific expression patterns.^20^ Recent studies have illuminated the pivotal roles that lncRNAs play in energy metabolism in cancer cells.^21^ LncRNAs, such as AGPG,^22^ MetaLnc9,^23^ LncRNA‐FEZF1‐AS1,^24^ and HULC,^25^ reprogram glucose metabolism by mediating post‐translational modifications of metabolic enzymes; LINK‐A,^26^ HISLA,^27^ GLCC1,^28^ and DRAIC^29^ regulate transcription factors that regulate genes related to aerobic glycolysis. However, the molecular regulatory network through which lncRNAs are involved in the reprogramming of glucose metabolism in HCC requires further investigation, particularly for lncRNAs that exhibit tumour‐specific expression. These lncRNAs have significant implications for developing novel therapeutic targets aimed at achieving metabolic modulation and show promise for clinical application in the treatment of HCC.

Y‐box binding protein 1 (YBX1) is a DNA/RNA binding protein that participates in various key biological processes such as transcriptional regulation, pre‐mRNA splicing, mRNA stability and stress responses.^30^ In the context of tumours, YBX1 is often overexpressed and is associated with aggressive phenotypes, increased cell proliferation, and metastasis.^31^, ^32^, ^33^, ^34^ YBX1 promotes epithelial–mesenchymal transition in sorafenib‐resistant HCC cells.^35^ Liquid–liquid phase separation of YBX1 is increased by circASH2, which leads to increased TPM4 transcript decay, thereby regulating metastasis by altering the tumour cytoskeletal structure.^36^ Emerging evidence suggests that YBX1 is involved in regulating cellular metabolism, including glucose metabolism and nucleotide metabolism, and represents a promising therapeutic target.^37^, ^38^ However, relatively little is known about the regulatory functions of YBX1 and lncRNAs in glucose metabolism in HCC.

In this study, we sought to investigate the effects of glucose‐related lncRNAs on HCC and clarify their mechanism to elucidate the regulatory network associated with glucose metabolic reprogramming. Critically, the potential of these lncRNAs as treatment targets was explored, offering novel therapeutic insights for HCC. Here, we revealed that LINC01419 expression is induced by glucose and that LINC01419 in turn promotes reprogramming of the glycolytic pathway and facilitates HCC progression. LINC01419 was reported to exhibit upregulated expression in HCC and is expected to serve as a biomarker,^39^ but previous research did not focus on its role in glucose metabolism. LINC01419 directly interacts with YBX1 and enhances its binding to its target gene PDK1, thereby increasing the mRNA stability of PDK1 and driving metabolic reprogramming to promote the proliferation and metastasis of HCC. Strikingly, LINC01419 is highly expressed in HCC, and a GalNAc‐conjugated siRNA targeting LINC01419 has shown promising therapeutic efficacy for HCC.

## 2 MATERIALS AND METHODS

### 2.1 Datasets for lncRNA expression analysis

Cohort 1 included 369 HCC patients in the RNA‐seq dataset from The Cancer Genome Atlas (TCGA‐LIHC, retrieved from the GDC Data Portal (https://portal.gdc.cancer.gov/)). Cohort 2 included 105 HCC patients (including 50 paired HCC tissues and normal tissues adjacent to the tumours (NATs)) obtained from the GepLiver project (http://www.gepliver.org/).^40^ Cohort 3 included 50 paired HCC tissues and NATs from the GSE77314 dataset, which was downloaded from the Gene Expression Omnibus (GEO, https://www.ncbi.nlm.nih.gov/geo/). Cohort 4 included 70 paired HCC tissues and NATs from the GSE144269 dataset, also downloaded from GEO.

### 2.2 Seahorse metabolic assays

Huh7 cells (10000 cells per well), PLC/PRF/5 (also known as PLC) cells (10000 cells per well) and SK‐Hep1 cells (12000 cells per well) were seeded in individual Seahorse XF96 cell culture plates and allowed to adhere overnight. A Seahorse XF Glycolysis Stress Test Kit (Agilent) was employed to determine the extracellular acidification rate (ECAR), and a Seahorse XF Cell Mito stress test kit (Agilent) was used to determine the oxygen consumption rate (OCR). All procedures were carried out in accordance with the manufacturer's instructions. The ECAR and OCR were quantitatively assessed utilizing Seahorse XFe96 Analyzers (Agilent). All data were analyzed using Seahorse Wave Pro software.

### 2.3 mRNA stability measurements

mRNA stability within the cells was achieved by incubating the cells with 5 µg/mL actinomycin D (MCE). Samples of the cells were harvested at the indicated time points, and total RNA was extracted for qPCR to measure the mRNA levels of PDK1. 18S rRNA was used for normalization.

### 2.4 In vivo assays

For the subcutaneous tumour formation assay in nude mice, Huh7 cells (3 × 10^6^) following NC or knockdown of LINC01419 by shRNA were subcutaneously injected into 6‐week‐old female BALB/c nude mice. For the in vivo rescue experiments, mice were injected subcutaneously with Huh7 cells (2 × 10^6^) following knockdown of LINC01419 and overexpression of YBX1/PDK1 (shNC and vector were used as the controls). For DCA treatment, mice were injected subcutaneously with Huh7 cells (1.5 × 10^6^) following vector or overexpression of LINC01419. Fourteen days after injection, each group of mice was randomly divided into two groups and treated with either saline or DCA (100 mg/kg) by gavage daily. The tumour volumes were measured every 2∼3 days and the tumour weights were assessed after the mice were sacrificed.

For the orthotopic xenograft assay, Huh7 cells stably expressing GFP‐luciferase (1.5 × 10^6^) following NC or knockdown of LINC01419 by shRNA were injected into the livers of 6‐week‐old female BALB/c nude mice. One month later, in vivo imaging was performed, after which the mice were euthanized, and the livers were harvested for further analysis.

For GalNAc‐siRNA treatment, the siRNA sequence targeting LINC01419 was CCTCAATTTCCATGGCAATAT with 2′‐O‐Me, FU and FC modifications. The GalNAc ligand was conjugated to the 3′ end of the sense strand of the siRNA using a triple GalNAc CPG analogue. GalNAc‐siRNA was obtained from Huzhou Hippo Biotechnology. Huh7 cells (5 × 10^6^) stably expressing GFP‐Luciferase were injected into 6‐week‐old male BALB/c nude mice to establish an orthotopic liver xenograft model. The mice were imaged weekly using the IVIS Lumina LT Series III In Vivo Imaging System (PerkinElmer) following anesthetization via isoflurane (RWD) inhalation after an intraperitoneal injection of 150 mg/kg d‐luciferin (Yeasen). Bioluminescence data were analyzed using Living Image software (PerkinElmer). One week after cell injection, the mice were randomly divided into two groups based on bioluminescence intensity and subsequently received weekly subcutaneous injections of GalNAc‐siNC or GalNAc‐siLINC0419 (5 mg/kg).

All the animal experiments were conducted in accordance with strict adherence to the institutional guidelines of the Institutional Animal Care and Use Committee of Fudan University Shanghai Cancer Center (permission number: FUSCC‐IACUC‐2022282), Shanghai, China.

### 2.5 Statistical analysis

Each experiment was conducted with three independent replicates, and the data are presented as the means ± standard deviations (SDs). For survival analysis, receiver operating characteristic curve analysis was employed to categorize the patients into two groups (with high/low LINC01419 expression), and differences in the Kaplan–Meier survival curves were assessed using the log‐rank test. Student's *t*‐test (two‐tailed) was applied for the comparison of means from two distinct samples, while one‐way analysis of variance was utilized for assessing differences among three or more samples. *p‐*values less than 0.05 were considered statistically significant (*p < 0.05, **p < 0.01, ***p < 0.001 and ****p < 0.0001). All statistical analyses were conducted using GraphPad Prism 9 (GraphPad Software) or IBM SPSS Statistics for Windows (version 26.0; Armonk). The details of the statistical analyses are provided in the figure legends.

## 3 RESULTS

### 3.1 Glucose‐induced LINC01419 facilitates tumour growth and metastasis of HCC cells

**FIGURE 1:**
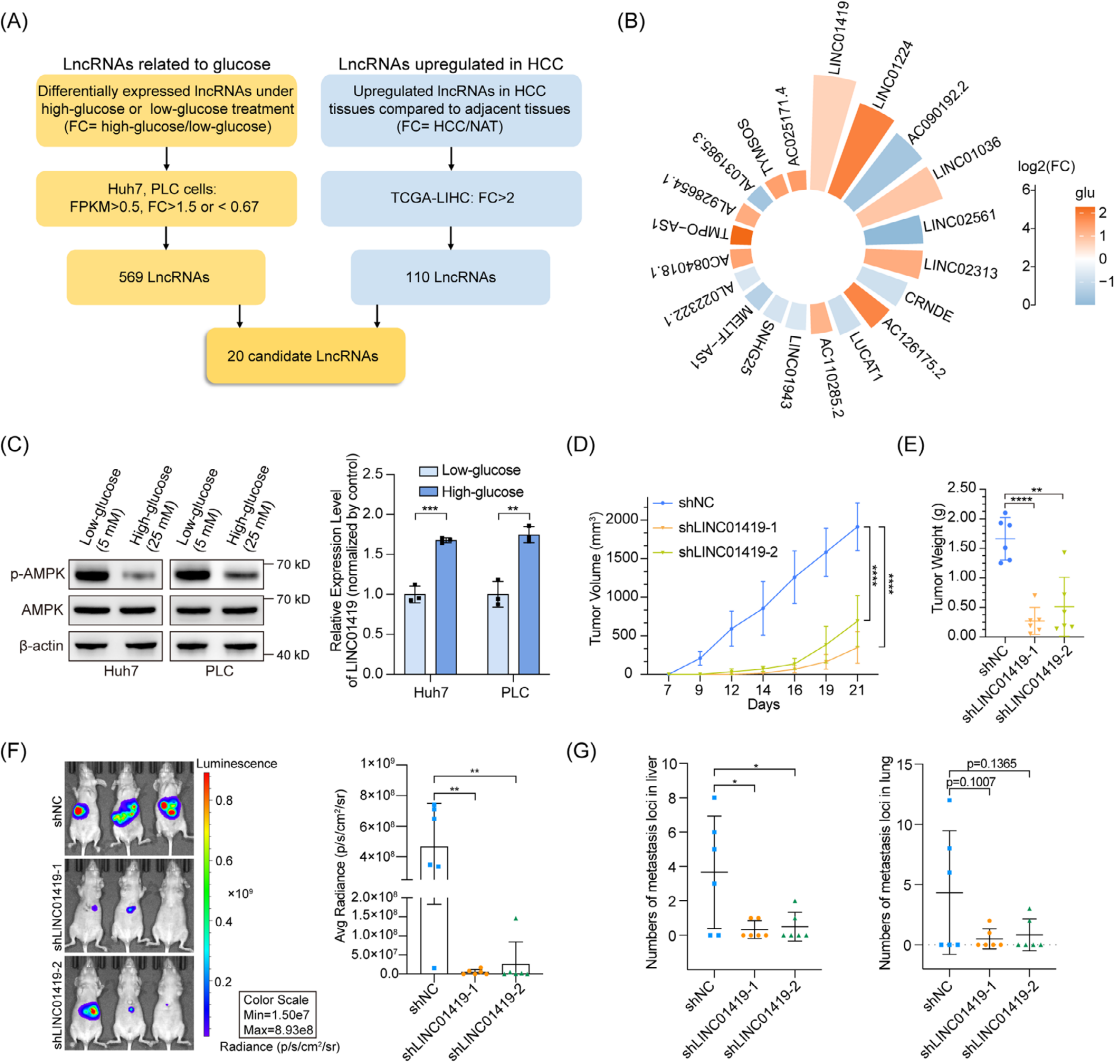
Glucose‐induced LINC01419 facilitates tumour growth and metastasis of HCC cells. (A) Flowchart of the process used to screen for glucose‐related lncRNAs that are upregulated in HCC cells (FC: fold change; low‐glucose: 5 mM, high‐glucose: 25 mM; NAT: normal tissue adjacent to the tumour). (B) Fold changes (FCs) in the expression of 20 candidate lncRNAs in HCC (FC, HCC/NAT) and HCC cells cultured in a medium with different glucose concentrations (5 and 25 mM) (FC, high‐glucose/low‐glucose). (C) Left: The expression levels of total AMPK protein and phosphorylated AMPK in Huh7 and PLC cells treated with low‐glucose (5 mM) or high‐glucose (25 mM) were detected by western blot. Right: Relative expression of LINC1419 in Huh7 and PLC cells cultured in low‐glucose (5 mM) or high‐glucose (25 mM) medium, as determined by qPCR. (D) The volume of tumours formed by Huh7 cells following NC or knockdown of LINC01419 by shRNA in the subcutaneous xenograft model in nude mice (*n* = 6). (E) Weights of tumours formed by Huh7 cells following NC or knockdown of LINC01419 by shRNA in the subcutaneous xenograft model of nude mice (*n* = 6). (F) Representative bioluminescence images of BALB/c nude mice (intrahepatically implanted with GFP‐luciferase‐transduced Huh7 cells following NC or knockdown of LINC01419 by shRNA) acquired before the mice were euthanized (left). The colour scale indicates the radiance emitted from these mice. Quantification of the average radiance for each group (right). (G) Statistical analysis of metastatic loci in images of H&E‐stained liver and lung tissues (*n* = 6). The data are presented as the means ± SDs. *p < 0.05, **p < 0.01, ***p < 0.001, ****p < 0.0001.

To investigate the role and therapeutic potential of glucose‐related lncRNAs in HCC, we performed conducted RNA‐sequencing (RNA‐seq) analysis of Huh7 and PLC cells cultured in low‐glucose (5 mM) or high‐glucose (25 mM) medium for 48 h and identified 569 lncRNAs whose expression changed significantly (FPKM > 0.5, fold change (=FPKM of high‐glucose/FPKM of low‐glucose) >1.5 or < 0.67) with alterations in glucose concentration (RNA‐seq dataset GSE234898). We then integrated these data with the data on differentially expressed lncRNAs (fold change > 2) in the LIHC cohort in TCGA (https://portal.gdc.cancer.gov/, TCGA‐LIHC dataset), and 20 candidate lncRNAs were identified as glucose‐related lncRNAs that are upregulated in HCC (Figure 1A; Table S1). Among these lncRNAs, LINC01419 was the most upregulated lncRNA with the highest abundance in HCC and was thus selected for further study (Figure 1B). As expected, RT‒qPCR assays demonstrated a notable upregulation of LINC01419 expression under high‐glucose conditions, and the activation of AMPK phosphorylation under low‐glucose conditions was used as a marker of successful glucose treatment (Figure 1C). Interestingly, LINC01419 was highly expressed in HCC tissues, exhibited almost no expression in NATs and was significantly associated with poorer overall survival (Figure S1A–E). These results indicated that LINC01419 might be a candidate oncogene involved in glucose metabolism in HCC.

In addition, we determined that LINC01419 was predominantly localized in the cytoplasm of HCC cells via nuclear and cytoplasmic fractionation and RNA fluorescence in situ hybridization (Figure S1F,G). The results of 3′ rapid amplification of cDNA ends (RACE) and 5′ RACE assays revealed that LINC01419 was a 1537‐nt long intergenic non‐coding RNA (Figure S1H).

Next, we explored the biological function of LINC01419 in vitro. Functional experiments with HCC cells following the knockdown or overexpression of LINC01419 consistently demonstrated the ability of LINC01419 to promote the proliferation, colony formation, migration and invasion of HCC cells (Figure S2A–H). To further validate the oncogenic role of LINC01419 in vivo, Huh7 cells stably transduced with two independent shLINC01419 constructs were subcutaneously injected into nude mice. LINC01419 deficiency markedly reduced both tumour growth and decreased the volume and weight of the tumours (Figure 1D,E; Figure S2I). Consistent with these findings, staining for the cell proliferation marker Ki67 was decreased in LINC01419‐deficient tumours (Figure S2J). Moreover, we established an orthotopic xenograft mouse model by injecting LINC01419‐knockdown cells into the livers of nude mice. In vivo imaging revealed that the knockdown of LINC01419 strongly suppressed in situ tumour growth (Figure 1F). The number of metastatic loci derived from LINC01419‐knockdown Huh7 cells was dramatically decreased in liver and lung sections, as shown by hematoxylin and eosin (H&E) staining (Figure 1G; Figure S2K).

Collectively, these findings suggest that glucose‐induced LINC01419 is associated with poor prognosis in HCC patients and promotes the growth and metastasis of HCC tumours. Previous reports have focused mainly on the molecular mechanisms of LINC01419 within the HCC cell nucleus,^39^, ^41^, ^42^ which prompted us to explore the regulatory mechanisms of LINC01419 within the cytoplasm in the context of glucose metabolism in HCC to identify new therapeutic targets.

### 3.2 YBX1 directly interacts with LINC01419 and acts as a downstream mediator

**FIGURE 2:**
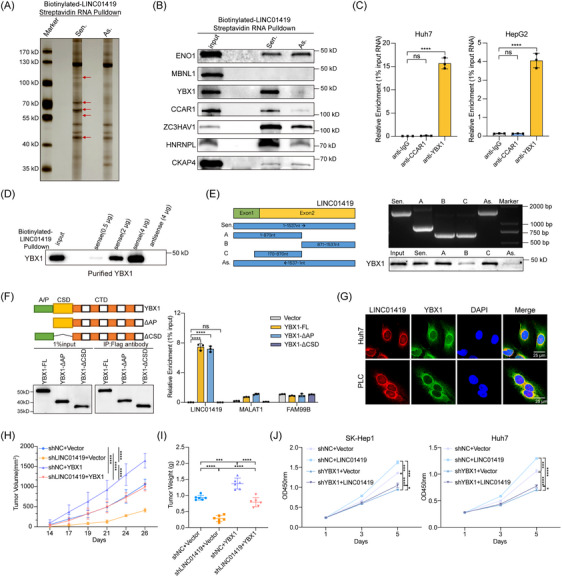
YBX1 directly interacts with LINC01419 and acts as a downstream mediator. (A) The LINC01419‐sense and LINC01419‐antisense probes were biotinylated and transcribed in vitro and were incubated with whole cell lysates of Huh7 cells for RNA pull‐down assays, LINC01419‐sense‐specific bands are marked with red arrows in the image of the PAGE gel after silver staining (Sen.: sense probe of LINC01419; As.: antisense probe of LINC01419). (B) Validation of proteins pulled down by the biotinylated sense or antisense probes of LINC01419 by western blot analysis. (C) RIP assays were performed using antibodies against CCAR1 or YBX1 in Huh7 and HepG2 cells, and qPCR assays were used to quantify the relative enrichment of LINC01419. (D) In vitro binding assays of biotinylated‐LINC01419 RNA with purified YBX1 protein were performed, and the enrichment of YBX1 was detected by western blot. (E) Schematic representation of LINC01419 truncations (left) and immunoblotting for YBX1 in samples pulled down with full‐length LINC01419, truncated LINC01419 fragments (A, 1–870 nt; B, 871–1537 nt; C, 170–870 nt) or the LINC01419 antisense probe. (F) Schematic representation of YBX1 truncations (AP: N‐terminal Ala/Pro‐rich domain; CSD: cold‐shock domain; CTD: C‐terminal domain) and the binding of truncated YBX1 fragments to LINC01419 was evaluated by RIP assays in cells transiently transfected with constructs containing Flag‐tagged full‐length (FL) YBX1 or truncated YBX1 fragments. Immunoprecipitation efficiency was measured by western blot (left) and the relative enrichment of LINC01419 was evaluated by qPCR (right). MALAT1 and FAM99B were used as negative control lncRNAs. (G) The colocalization of LINC01419 and YBX1 in Huh7 and PLC cells was evaluated by fluorescence staining. Scale bar = 25 µm. (H) Mice were injected subcutaneously with 2 × 10^6^ Huh7 cells following the knockdown of LINC01419 and the overexpression of YBX1 (shNC and vector as the controls), and the tumour volume was measured every 2 to 3 days after tumour formation. Tumour volume (left) and image of subcutaneous tumour tissues from individual groups (right) (*n* = 6). (I) Weights of tumours formed by Huh7 cells following the indicated treatment in a subcutaneous xenograft model in nude mice (*n* = 6). (J) CCK‐8 assays of SK‐Hep1 and Huh7 cells with knockdown of YBX1 by shRNA and stable overexpression of LINC01419 (shNC and vector as the controls). The data are presented as the means ± SDs. *p < 0.05, **p < 0.01, ***p < 0.001, ****p < 0.0001; differences with *p* > 0.05 were considered non‐significant (ns).

To delve into the molecular mechanism through which LINC01419 functions as an oncogenic lncRNA in the cytoplasm of HCC cells, we performed a biotin‐labelled RNA pulldown assay followed by mass spectrometry (MS) to identify the proteins that may interact with LINC01419 in HCC cells. The results from three independent LINC01419 pulldown experiments consistently revealed several different bands in the sliver‐stained PAGE gel, and the mass spectrometric data revealed that several proteins were pulled down with LINC01419 RNA (Figure 2A). Thirteen potential LINC01419‐interacting proteins were identified based on the criteria of at least five unique peptides and a fold change in sense/antisense areas greater than or equal to five (Figure S3A). Given that LINC01419 was expressed predominantly in the cytoplasm and that this localization was associated with a specific function, proteins localized in the cytoplasm were evaluated by western blot. Among the investigated proteins, CCAR1 and YBX1 were confirmed to interact with LINC01419 (Figure 2B). However, subsequent RNA immunoprecipitation (RIP) experiments indicated that YBX1 protein and LINC01419 coprecipitated with an antibody targeting YBX1, whereas no enrichment of LINC01419 was detected with the anti‐CCAR1 antibody, suggesting a specific interaction between YBX1 and LINC01419 (Figure 2C; Figure S3B). Additional in vitro RNA‒protein binding assays using the purified YBX1 protein with biotin‐labeled LINC01419 demonstrated that LINC01419 pulled down YBX1 in a concentration‐dependent manner, confirming the direct interaction of LINC01419 with YBX1 (Figure 2D). To identify the region of LINC01419 that binds to the YBX1 protein, we constructed three truncated fragments of LINC01419 (fragment A:1–870 nt, fragment B: 871–1537 nt and fragment C: 170–870 nt) on the basis of predicted RNA secondary structure from the RNAfold web server (http://rna.tbi.univie.ac.at/cgi‐bin/RNAWebSuite/RNAfold.cgi) (Figure S3C) and conducted an RNA pulldown assay. The results showed that YBX1 bound mainly to the fragment C of LINC01419 (Figure 2E). We next constructed Flag‐tagged full‐length YBX1 and truncations of YBX1 based on its functional domains. Further, RIP assays revealed that LINC01419 interacted primarily with the cold shock domain (CSD) of YBX1, with no differential enrichment observed for the other negative control (NC) lncRNAs (Figure 2F). Consistent with the direct interaction of LINC01419 with YBX1, we observed their partial colocalization in HCC cells (Figure 2G). YBX1 plays an essential role in several cancers.^38^, ^43^, ^44^ We found that the inhibition of cell proliferation, migration, and invasion due to LINC01419 knockdown was significantly reversed by YBX1 overexpression. (Figure S4A–E). Consistently, overexpression of YBX1 countered the inhibitory effect of LINC01419 knockdown on tumour growth in vivo (Figure 2H,I; Figure S4F–H). In addition, the knockdown of YBX1 significantly abrogated the capacity of LINC01419 overexpression to promote cell proliferation, migration, and invasion (Figure 2J; Figure S4I–K), further indicating that YBX1 is a key downstream mediator of LINC01419.

### 3.3 LINC01419 and YBX1 reprogram glucose metabolism by increasing PDK1 expression

**FIGURE 3:**
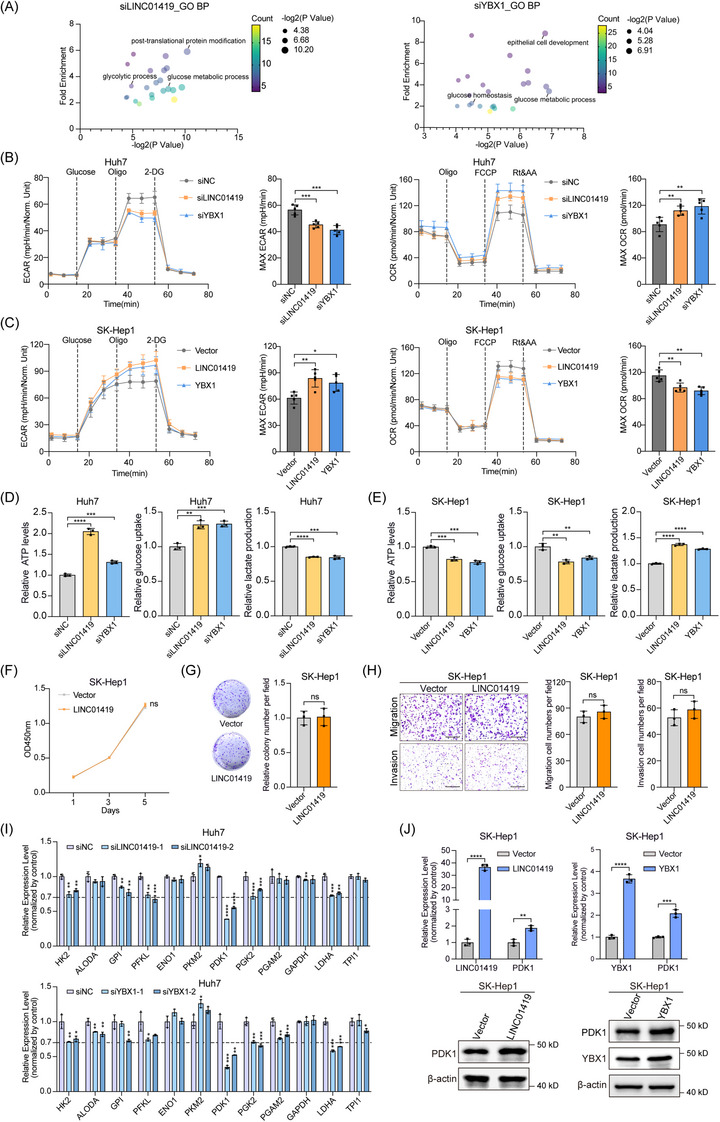
LINC01419 and YBX1 reprogram glucose metabolism by increasing PDK1 expression. (A) Gene Ontology biological process analysis results of downregulated gene sets with FC < 0.67 in Huh7 cells subjected to siRNA‐mediated knockdown of LINC01419 (left) or YBX1 (right). (B, C) Extracellular acid ratio (ECAR) and oxygen consumption ratio (OCR) in Huh7 cells following knockdown of LINC01419 or YBX1 by siRNA (B) and in SK‐Hep1 cells following stable overexpression of LINC01419 or YBX1 (C), measure with a Seahorse metabolic analyzer. The MAX ECAR/OCR is shown on the right side. (D, E) Relative ATP levels, glucose uptake and lactate production in Huh7 cells following knockdown of LINC01419 or YBX1 by siRNA (D) and in SK‐Hep1 cells following stable overexpression of LINC01419 or YBX1 (E). (F–H) CCK‐8 (F), colony formation (G), migration and invasion (H) assays of SK‐Hep1 cells following stable overexpression of LINC01419 cultured in medium containing galactose instead of glucose. Scale bar = 200 µm. (I) Relative expression levels of glycolytic enzymes in Huh7 cells following knockdown of LINC01419 or YBX1, as determined by qPCR. (J) qPCR and western blot analyses of the expression of PDK1 in SK‐Hep1 cells following stable expression of LINC01419 or YBX1. The data are presented as the means ± SDs. *p < 0.05, **p < 0.01, ***p < 0.001, ****p < 0.0001; differences with *p* > 0.05 were considered non‐significant (ns).

To clarify the mechanism underlying the ability of the LINC01419‐YBX1 complex to promote HCC growth and metastasis, we next conducted RNA‐sequencing following individual knockdown of LINC01419 and YBX1 to reveal the possible downstream biological processes in HCC cells (Table S2). Gene Ontology biological process analysis demonstrated that both LINC01419 and YBX1 might affect glucose metabolism (Figure 3A). Consequently, we utilized Seahorse assays to assess glycolysis and mitochondrial respiration. The results suggested that LINC01419 and YBX1 indeed regulate glucose metabolism. Knocking down LINC01419 or YBX1 significantly reduced the ECAR and increased the maximal OCR in Huh7 and PLC cells (Figure 3B; Figure S5A), whereas the overexpression of these factors had the opposite effects in SK‐Hep1 cells (Figure 3C). Accordingly, we found elevated cellular ATP levels and glucose uptake, along with decreased extracellular lactate levels, with knockdown of LINC01419 or YBX1, whereas overexpression of either resulted in the opposite changes (Figure 3D,E; Figure S5B). Furthermore, we investigated the involvement of glycolysis in the ability of LINC01419 to promote HCC growth and metastasis. We substituted glucose in the cell culture medium with galactose and found that the reduction in the glycolytic flux strongly suppressed the promotion of cell growth, migration and invasion induced by LINC01419 overexpression (Figure 3F–H; Figure S5C–E), indicating that the oncogenic function of LINC01419 was dependent on the glycolytic process. To gain further insight into the mechanism through which LINC01419 regulates glycolysis, we examined the effects of LINC01419 on the expression of key glycolytic enzymes. Interestingly, the expression of PDK1 was most significantly downregulated by either LINC01419 or YBX1 knockdown in Huh7 and PLC cells (Figure 3I; Figure S6A). PDK1 is an enzyme crucial for metabolic reprogramming, as it inhibits the activity of PDH and facilitates the conversion of pyruvate to lactate, a process recognized as aerobic glycolysis or the Warburg effect.^12^ Elevated levels of PDK1 expression have been implicated in tumour progression, metastasis and the development of resistance to chemotherapy.^13^, ^14^, ^15^, ^16^ For further clarification, we measured the RNA and protein expression levels of PDK1 and observed consistent downregulation of PDK1 upon LINC01419 or YBX1 knockdown in Huh7 and PLC cells (Figure S6B,C). Conversely, the overexpression of either of these factors significantly upregulated PDK1 expression (Figure 3J; Figure S6D).

### 3.4 LINC01419 reprograms the glycolytic pathway by recruiting YBX1 to enhance PDK1 mRNA stability

**FIGURE 4:**
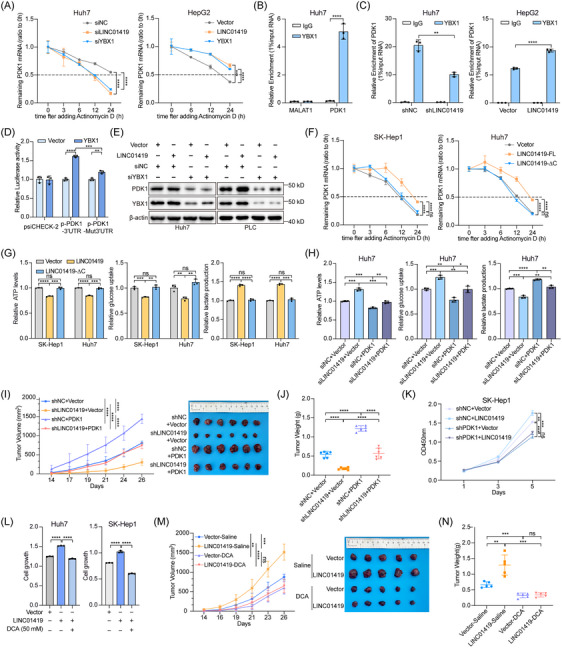
LINC01419 reprograms the glycolytic pathway by recruiting YBX1 to enhance PDK1 mRNA stability. (A) Relative RNA expression of PDK1 in Huh7 cells following knockdown of LINC01419 or YBX1 (left) and in HepG2 cells following stable overexpression of LINC01419 or YBX1 (right) at different time points after treatment with actinomycin D (5 µg/mL) was detected by qPCR. (B) RIP assays were performed using antibodies against YBX1 in Huh7 cells. The relative enrichment of PDK1 was evaluated by qPCR, and MALAT1 was used as a negative control. (C) Enrichment of YBX1 in the region of PDK1 in Huh7 cells (left) following knockdown of LINC01419 (shNC as the control) and in HepG2 cells (right) following stable overexpression of LINC01419 (Vector as the control) was detected by RIP‒qPCR. (D) Relative luciferase activity of psi‐CHECK‐2 containing the PDK1‐3′UTR (p‐PDK1‐3′UTR) or PDK1‐△3′UTR (p‐PDK1‐Mut3’UTR) sequence in Huh7 cells following stable overexpression of YBX1 (Vector as the control). (E) Western blot analysis of PDK1 and YBX1 protein expression in Huh7 and PLC cells with the indicated treatments. (F) Relative RNA expression of PDK1 in SK‐Hep1 and Huh7 cells following stable overexpression of full‐length and truncated LINC01419 (LINC01419‐∆C) at different time points after treatment with actinomycin D (5 µg/mL) was detected by qPCR. (G) Relative ATP levels (left), glucose uptake (middle) and lactate production (right) in SK‐Hep1 and Huh7 cells following stable overexpression of full‐length and truncated LINC01419 (LINC01419‐∆C). (H) Relative ATP levels (left), glucose uptake (middle) and lactate production (right) in Huh7 cells with the indicated treatments. (I) Mice were injected subcutaneously with 2 × 10^6^ Huh7 cells following knockdown of LINC01419 and overexpression of PDK1 (shNC and vector as the controls), tumour volume was measured every 2 to 3 days after tumour formation. Tumour volumes (left) and images of subcutaneous tumour tissues from individual groups (right) (*n* = 6). (J) Weights of tumours formed by Huh7 cells following the indicated treatments in a subcutaneous xenograft model in nude mice (*n* = 6). (K) CCK‐8 assays of SK‐Hep1 cells following knockdown of PDK1 by shRNA stable and overexpression of LINC01419 (shNC and vector as the controls). (L) CCK‐8 assays of Huh7 and SK‐Hep1 cells following overexpression of LINC01419 and treatment with DCA (50 mM) for 24 h. (M) Mice were injected subcutaneously with 1.5 × 10^6^ Huh7 cells following overexpression of LINC01419 (vector as the control). Fourteen days after injection, each group of mice was randomly divided into two groups and were treated with saline or DCA (100 mg/kg) by gavage daily (*n* = 5). Tumour volumes (left) and image of subcutaneous tumour tissues from individual groups (right). (N) Tumour weights of each group are shown (*n* = 5). The data are presented as the means ± SDs. *p < 0.05, **p < 0.01, ***p < 0.001, ****p < 0.0001; differences with *p* > 0.05 were considered non‐significant (ns).

On the basis of the increased PDK1 expression and glycolysis induced by LINC01419 and YBX1, we further explored the specific regulatory mechanism underlying these alterations. The results of the luciferase reporter assay demonstrated that the promoter activity of PDK1 remained unaltered by knockdown or overexpression of either LINC01419 or YBX1, indicating that these genes do not affect the transcription of PDK1 (Figure S7A). Following actinomycin D treatment, knockdown of LINC01419 or YBX1 accelerated but overexpression of LINC01419 or YBX1 retarded PDK1 degradation in HCC cells (Figure 4A; Figure S7B). These results indicated that LINC01419 and YBX1 facilitated PDK1 expression by stabilizing PDK1 mRNA, a mechanism that aligns with the predominantly cytoplasmic localization of LINC01419, revealing its role in post‐transcriptional regulation. YBX1 can bind to and stabilize its target mRNAs as an RNA‐binding protein.^45^ Additionally, the 3′ UTRs of mRNAs are often recognized and bound by miRNAs or RBPs to regulate their stability.^46^ YBX1 RIP‒seq revealed a notable peak in the 3′UTR of PDK1 mRNA in Huh7 cells, indicating that YBX1 may directly bind to the 3′UTR of PDK1 mRNA, thereby enhancing its stability (Figure S7C). Moreover, the RIP‒qPCR results revealed that YBX1 can bind to PDK1 mRNA in Huh7 cells (Figure 4B). To assess the role of LINC01419 in the binding of YBX1 to PDK1 mRNA, we performed a RIP assay. Our findings indicated that the interaction between YBX1 and PDK1 mRNA was attenuated by the knockdown of LINC01419, while it was enhanced by the overexpression of LINC01419 (Figure 4C; Figure S7D,E). Furthermore, we inserted the PDK1‐3′UTR sequence or the PDK1‐Δ3’UTR sequence with a deleted region into the fluorescent reporter vector psi‐CHECK‐2. The luciferase activity of the PDK1‐3′UTR reporter was notably increased with YBX1 expression, whereas the effect on the PDK1‐Δ3’UTR reporter was diminished, highlighting the critical role of the PDK1 3′UTR in the binding of YBX1 to PDK1 mRNA (Figure 4D). Subsequent RNA pulldown and in vitro binding experiments revealed that YBX1 could directly bind to PDK1 3′UTR (Figure S7F). In addition, the knockdown of YBX1 inhibited the upregulation of PDK1 caused by LINC01419 overexpression in Huh7 and PLC cells (Figure 4E). These results indicated that LINC01419 enhanced the binding of YBX1 to PDK1 mRNA, thereby regulating PDK1 mRNA stability in a YBX1‐dependent manner. We further constructed a vector containing LINC01419 lacking the YBX1‐binding region (LINC01419‐∆C). Functional experiments revealed that this truncated form of LINC01419, which was unable to interact with YBX1, lost its ability to stimulate the proliferation, migration and invasion of HCC cells (Figure S7G–J). Moreover, the results of the mRNA stability assays revealed that the truncated LINC01419 did not increase PDK1 mRNA stability (Figure 4F; Figure S7K,L). Similarly, disruption of the interaction between LINC01419 and YBX1 eliminated the effects of LINC01419 on glucose metabolism, as shown by ATP, glucose and lactate levels (Figure 4G). These findings suggested that the interaction of LINC01419 and YBX1 was necessary for the observed increase in YBX1 binding to and stabilization of PDK1 mRNA.

To investigate whether PDK1 is required for the LINC01419‐mediated promotion of HCC growth and metastasis, functional rescue experiments were performed. Ectopic expression of PDK1 reversed the decreases in the growth, colony formation, cell migration and invasion abilities of LINC01419‐knockdown cells (Figure S8A–E). Consistent with this conclusion, the changes in glucose metabolism markers observed upon knockdown of LINC01419 were also reversed by overexpression of PDK1 (Figure 4H). In addition, overexpression of PDK1 reversed the suppressive effect of LINC01419 knockdown on tumour growth in vivo (Figure 4I,J; Figure S8F,G). The impact of LINC01419 overexpression on cell proliferation, migration, and invasion was reversed by PDK1 knockdown, further suggesting that PDK1 serves as a functional target of LINC01419 in HCC cells (Figure 4K; Figure S8H–K). Additionally, we employed two PDK1 inhibitors, JX06^47^ and DCA,^48^ for cellular and metabolic assays. As expected, both JX06 and DCA attenuated the enhancements in cell growth and colony formation ability caused by LINC01419 overexpression (Figure 4L; Figure S9A–C). The altered metabolic phenotypes of glucose uptake, ATP production and lactate production in LINC01419‐overexpressing cells were similarly reversed by treatment with the PDK1 inhibitor JX06 (Figure S9D,E). The PDK1 inhibitor DCA has long been explored as a possible therapeutic candidate for various medical conditions.^18^, ^19^ It has been explored as an experimental medication for the treatment of type 2 diabetes mellitus, myocardial ischemia and heart failure, and most recently in clinical trials for cancer.^48^, ^49^ Hence, we administered DCA (100 mg/kg) or saline daily by gavage in a subcutaneous tumour model in nude mice.^50^ The results demonstrated that DCA effectively suppressed the tumour growth triggered by overexpression of LINC01419 (Figure 4M,N; Figure S9F), further indicating that PDK1 is the major downstream effector of LINC0419. Overall, our findings reveal the critical role of the LINC01419/YBX1‐PDK1 axis in promoting HCC progression through metabolic reprogramming.

### 3.5 DNA demethylation and Yin‐Yang 1 (YY1) are required for the transcriptional activation of LINC01419 under high‐glucose conditions in HCC cells

**FIGURE 5:**
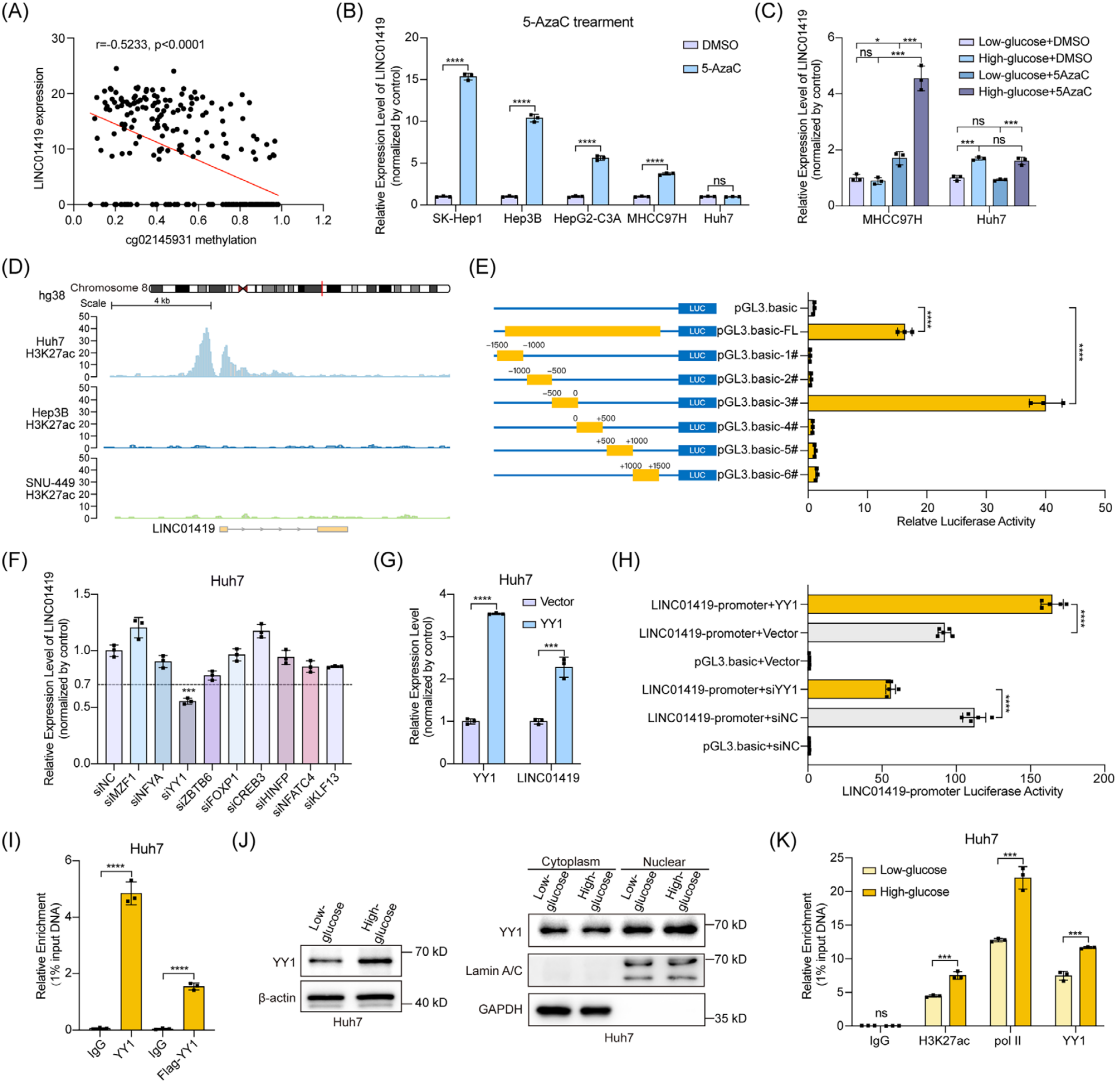
DNA demethylation and Yin‐Yang 1 (YY1) are required for the transcriptional activation of LINC01419 under high‐glucose conditions in HCC cells. (A) Correlation between the DNA methylation levels of cg02145931 and LINC01419 expression in the TCGA‐LIHC dataset (*n* = 236 liver cancer tissues in the LIHC cohort, Pearson correlation analysis). (B) The relative expression of LINC01419 in HCC cells after 5‐AzaC (10 µM) treatment for 72 h was determined by qPCR. (C) The relative expression of LINC01419 in MHCC97H and Huh7 cells cultured in low‐glucose (5 mM) or high‐glucose (25 mM) medium and treated with DMSO or 5‐AzaC (10 µM) for 72 h was determined by qPCR. (D) Alignment of ChIP‒seq data from the HCC cell lines Huh7, Hep3B, and SNU‐449 to the hg38 genome revealed enrichment of H3K27ac in the promoter of LINC01419. (E) Identification of the core region in the LINC01419 promoter. Diagrams of the full‐length LINC01419 promoter and the truncated promoter fragments (left); luciferase activity of the full‐length and truncated promoter fragments in HEK‐293T cells (right). (F) Relative expression of LINC01419 in Huh7 cells following knockdown of different transcription factors with a siRNA mixture. (G) Relative RNA expression of YY1 and LINC01419 in Huh7 cells following overexpression of YY1 (Vector as the control). (H) Relative promoter activity of LINC01419 in HEK‐293T cells with knockdown or overexpression of YY1. (I) ChIP‒qPCR assays (with an anti‐YY1 antibody or anti‐Flag‐YY1, with anti‐IgG as the negative control) were performed to evaluate the binding of YY1 to the promoter of LINC01419 in Huh7 cells. (J) The protein level of YY1 (left) and the nuclear‐cytoplasmic distribution of YY1 (right) in Huh7 cells cultured under low‐glucose (5 mM) and high‐glucose (25 mM) conditions were evaluated by western blot analysis. GAPDH was used as a cytoplasmic internal control, Lamin A/C was used as a nuclear internal control. (K) The enrichment of H3K27ac, RNA pol II and YY1 in the LINC01419 promoter region in Huh7 cells cultured under low‐glucose (5 mM) and high‐glucose (25 mM) conditions was detected by ChIP‒qPCR. The data are presented as the means ± SDs. *p < 0.05, ***p < 0.001, ****p < 0.0001; differences with *p* > 0.05 were considered non‐significant (ns).

Next, we explored the upstream mechanisms regulating the expression of LINC01419. LINC01419 was not detected in normal human tissues, with the exception of the testis (Figure S10A), but was expressed in several types of cancers, with particularly high expression in HCC (Figure S10B), suggesting that LINC01419 is a cancer‐specific lncRNA. To investigate the abnormal activation of LINC01419 within HCC cells, we utilized MethPrimer (http://www.urogene.org/methprimer) to forecast the presence of CpG islands in the promoter region of LINC01419 (Figure S10C). The DNA methylation chip sequencing data of HCC samples from TCGA indicated that a methylation probe was identified within the promoter region of LINC01419, and the extent of DNA methylation at this site (cg02145931) exhibited a substantial inverse correlation with the expression level of LINC01419 (Figure 5A). The expression of LINC01419 in multiple HCC cell lines with relatively low basal expression levels was significantly increased by 5‐aza‐2′‐deoxycytidine (5‐AzaC) treatment, whereas there was little change in LINC01419 expression in Huh7 cells, which have high LINC01419 expression (Figure 5B), implying the role of DNA methylation in the transcriptional inhibition of LINC01419. Intriguingly, high‐glucose concentrations did not increase LINC01419 expression in MHCC97H cells as they did in Huh7 cells, whereas high‐glucose concentrations induced the expression of LINC01419 in response to 5‐AzaC treatment (Figure 5C). These results suggested that DNA hypomethylation in the promoter region was necessary for LINC01419 transcription and was a prerequisite for glucose‐induced LINC01419 expression in HCC cells.

We further explored the mechanism behind the specific upregulation induced by high‐glucose concentrations in HCC cells. We analyzed histone modifications in the LINC01419 promoter in HCC cells and detected strong enrichment of H3K27ac peaks in the promoter region of LINC01419 in Huh7 cells, which have high levels of LINC01419; conversely, no peaks were detected in Hep3B and SNU‐449 cells, which have extremely low LINC01419 expression (Figure 5D). A luciferase reporter assay was subsequently conducted to pinpoint the core region in the LINC01419 promoter. Measurement of the luciferase activities arising from the full‐length LINC01419 promoter as well as a series of truncated LINC01419 promoters revealed that the luciferase activity of the promoter fragment encompassing nt‐500∼0 was considerably greater compared with other fragments (Figure 5E). Examination of this core promoter region via the UCSC and JASPAR databases uncovered multiple transcription factor‐binding sites. We silenced the transcription factors with high scores and elevated expression in HCC and observed significantly decreased expression of LINC01419 upon knockdown of YY1 in Huh7 cells, while overexpression of YY1 induced the expression of LINC01419 (Figure 5F,G). Consistent with these observations, the promoter activity of LINC01419 was reduced by the knockdown of YY1 and enhanced by the overexpression of YY1 (Figure 5H), indicating that YY1 might be a key regulator for the glucose‐induced elevation of LINC01419 expression in HCC cells. Subsequent chromatin immunoprecipitation (ChIP) experiment indicated that YY1 was significantly enriched in the promoter region of LINC01419 (Figure 5I). Additionally, YY1 enrichment in the promoter region of LINC01419 was also observed in previously reported public YY1‐ChIP‐seq data (Figure S10D). YY1 plays a role in regulating a variety of genes across diverse biological processes and is implicated in various diseases.^51^, ^52^, ^53^ It has been documented that YY1 is vital for the transcription initiation of transposable elements.^54^ Given that LINC01419 expression was upregulated by high‐glucose concentrations, we investigated the status of YY1 under high‐glucose conditions. Interestingly, the expression of YY1 was notably elevated, with a concomitant rise in the nuclear localization of YY1 under high‐glucose conditions compared with low‐glucose conditions (Figure 5J; Figure S10E). We also performed a ChIP assay in Huh7 cells cultured both in low‐ and high‐glucose conditions, observing increased occupancy of H3K27ac, Pol II, and YY1 in the promoter of LINC01419 under high‐glucose conditions (Figure 5K).

Overall, these findings indicate that hypomethylation of the promoter region and YY1 are necessary for the transcriptional activation of LINC01419 under high‐glucose conditions in HCC cells.

### 3.6 Administration of GalNAc‐siLINC01419 effectively inhibits the growth and metastasis of orthotopic tumour xenografts in vivo

**FIGURE 6:**
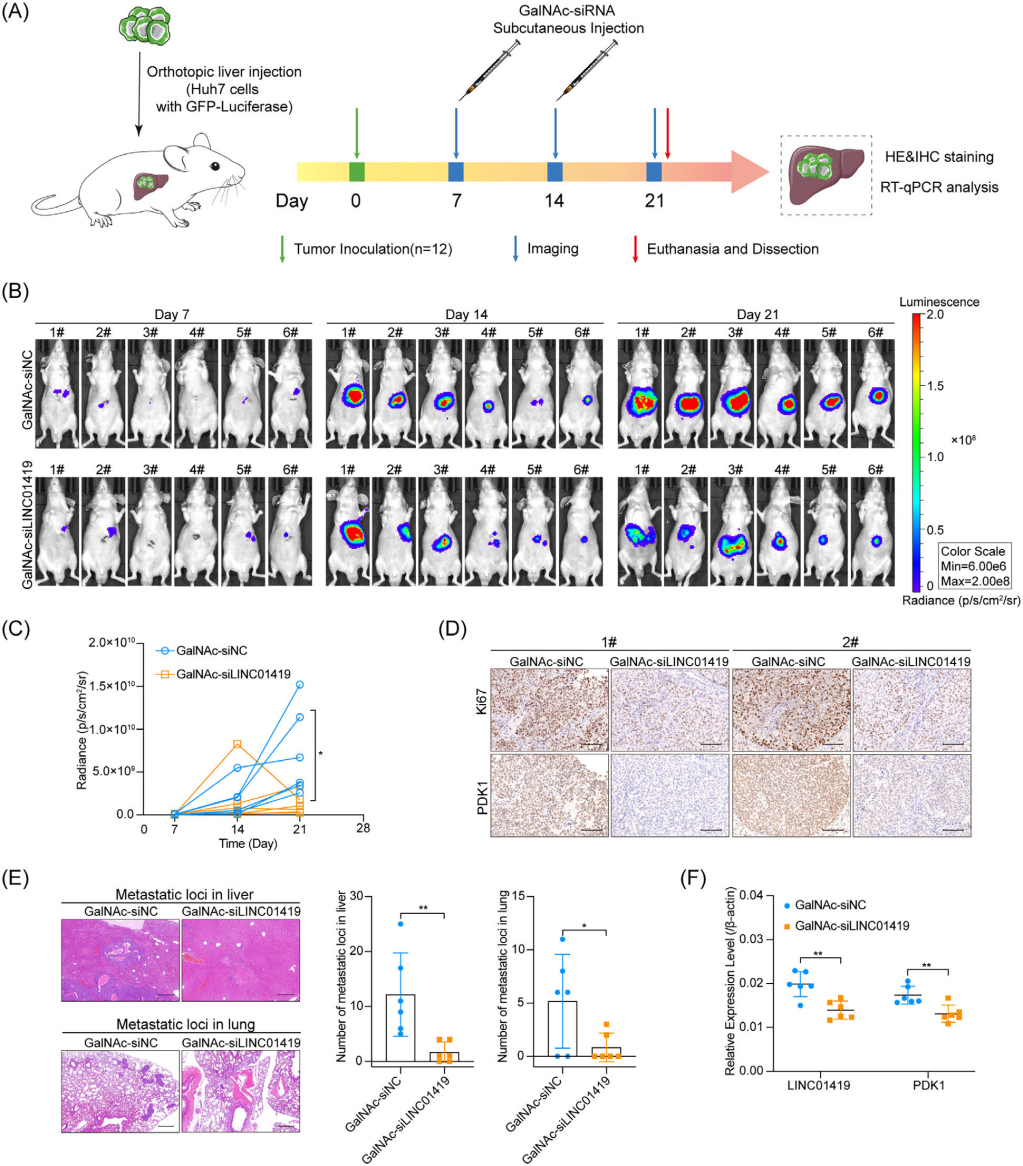
The Administration of GalNAc‐siLINC01419 effectively inhibited the growth and metastasis of orthotopic tumour xenografts in vivo. (A) Schematic diagram of GalNAc‐siLINC01419 treatment of orthotopic liver tumours in mice. Huh7 cells stably expressing GFP‐Luciferase were orthotopically injected into the livers of BALB/c nude mice. After 7 days, the mice were randomly divided into two groups (*n* = 6) and subcutaneously administered GalNAc‐siNC or GalNAc‐siLINC01419 at a dose of 5 mg/kg weekly. The size of in situ tumours was monitored by weekly in vivo bioluminescence imaging. On day 21, the mice were performed with euthanasia after in vivo imaging and the livers of mice were used for immunohistochemistry (IHC) staining, H&E staining and expression analysis. (B) Weekly in vivo bioluminescence imaging revealing the tumour growth of mice treated with GalNAc‐siNC or GalNAc‐siLINC01419. (C) Statistical analysis of the average radiance determined by in vivo imaging (*n* = 6). (D) Representative images of IHC staining for Ki67 and PDK1 in the GalNAc‐siNC‐ and GalNAc‐siLINC01419‐treated groups. Scale bar = 100 µm. (E) Representative images of H&E‐stained samples from the GalNAc‐siNC‐ or GalNAc‐siLINC01419‐treated groups (left) and statistical analyses of metastatic loci (right). Scale bar = 500 µm. (F) Relative expression of LINC01419 and PDK1 in liver tumours in the orthotopic xenograft mouse model, as determined by qPCR. The data are presented as the means ± SDs. *p < 0.05, **p < 0.01.

Various RNA‐based therapeutics employing primarily antisense oligonucleotides (ASOs) and siRNAs have been developed, with several gaining FDA approval; these agents have emerged as promising treatments for cancers and a variety of other diseases. However, the mode and efficiency of RNA delivery represent pivotal challenges for successful RNA‐based therapeutics. GalNAc serves as a high‐affinity ligand of the hepatocyte‐specific asialoglycoprotein receptor, which can facilitate siRNA uptake into hepatocytes through clathrin‐mediated endocytosis and thus has great advantages for liver‐targeted delivery. Given that LINC01419 is predominantly expressed in HCC cells and not in normal hepatocytes, suggesting that administering GalNAc‐siLINC01419 could serve as a potential therapeutic approach for HCC. Huh7 cells stably expressing GFP‐luciferase were orthotopically injected into the livers of BALB/c nude mice to establish an orthotopic xenograft model of HCC. The mice received subcutaneous injections of either GalNAc‐siLINC01419 or GalNAc‐siNC (*n* = 6 mice/group) at a dose of 5 mg/kg twice, and in vivo bioluminescence imaging was performed to track tumour growth weekly (Figure 6A). Notably, in vivo imaging analyses demonstrated that the administration of GalNAc‐siLINC01419 obviously inhibited the growth of tumour xenografts in the mice (Figure 6B,C; Figure S11A). Consistent with this observation, the immunohistochemistry (IHC) staining in xenografts showed decreased cell proliferation marker Ki67 and PDK1 expression upon the knockdown of LINC01419 with GalNAc‐siLINC01419 (Figure 6D). Moreover, H&E staining revealed fewer intrahepatic and lung metastases in the group treated with GalNAc‐siLINC01419 (Figure 6E). Consistent with expectations, the mRNA and protein levels of PDK1 were notably decreased following the treatment of GalNAc‐siLINC01419 (Figure 6F; Figure S11B). In summary, GalNAC‐siLINC01419 markedly suppressed the growth and metastasis of orthotopic tumour xenografts in vivo, suggesting that it could serve as a valuable targeted therapeutic approach for HCC patients with high levels of LINC01419 expression.

## 4 DISCUSSION

**FIGURE 7:**
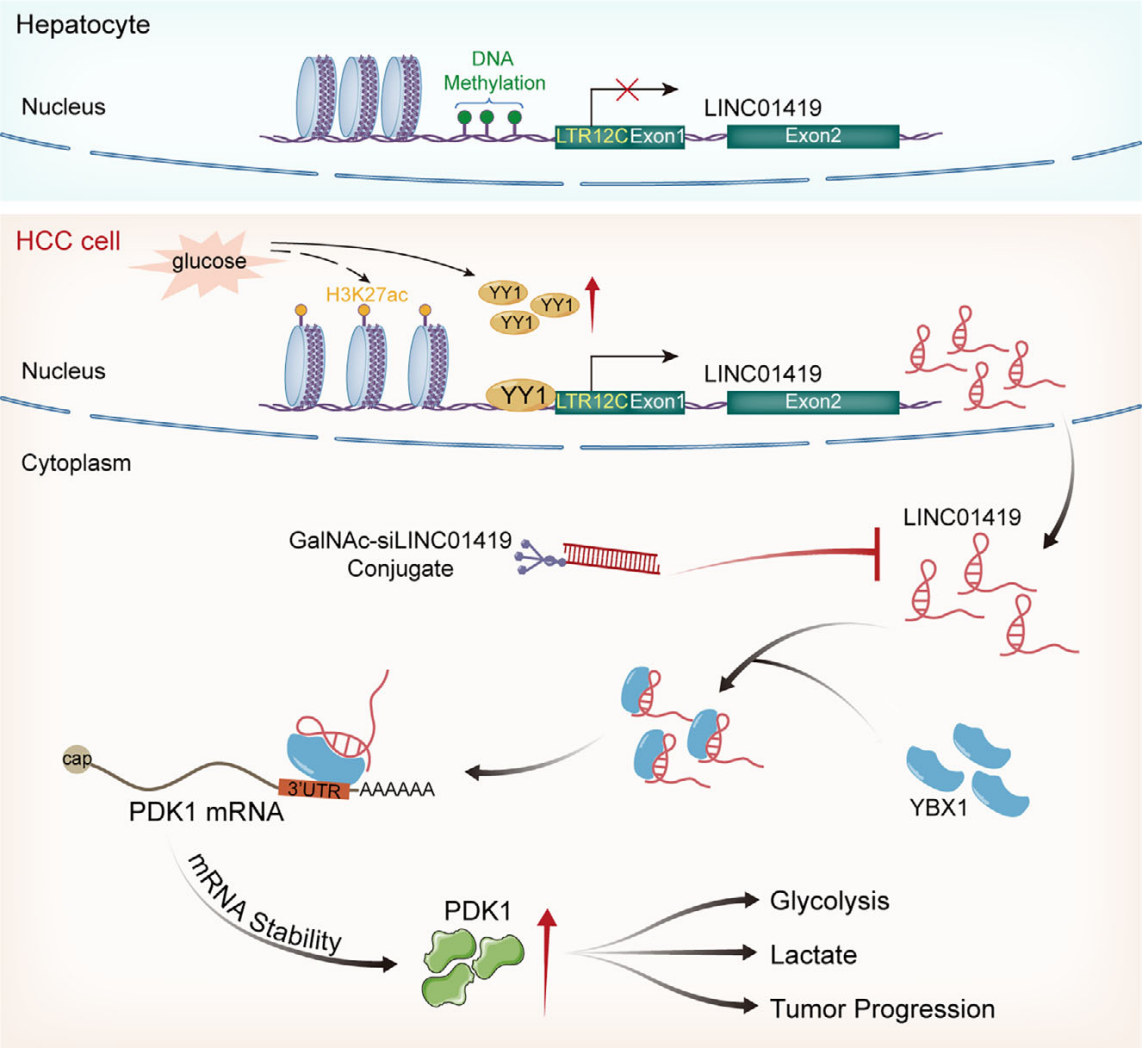
Schematic overview of the mechanism of LINC01419 in glycolytic pathway reprogramming in HCC. Cancer‐specific LINC01419 expression is induced by the transcription factor YY1 with increasing glucose concentrations and participates in the progression of HCC by regulating the glycolytic pathway. Mechanistically, LINC01419 directly binds to YBX1 in the cytoplasm and recruits it to PDK1 mRNA, thus enhancing the mRNA stability of PDK1. Notably, a GalNAc‐conjugated siRNA targeting LINC01419 could serve as a potential targeted therapeutic strategy for patients with advanced HCC.

Metabolic reprogramming provides most of the energy required for the initiation and progression of cancer. Additionally, the glycolytic activity of HCC cells is much greater than that of normal hepatocytes.^55^ Currently, targeted therapy and immunological therapy are the main treatment options for advanced HCC, but their efficacy is limited.^56^, ^57^ Targeting key molecules involved in metabolic reprogramming in HCC cells is likely to provide substantial benefits for patients with advanced HCC. Previous investigations have revealed the oncogenic mechanisms of LINC01419 within the context of HCC, such as binding to RNF169 to promote the initiation of DNA homologous recombination repair,^41^ increasing ZIC1 promoter methylation to inhibit ZIC1 expression and activate the PI3K/Akt signalling pathway,^42^ and activating the NDRG1 promoter to induce cell proliferation and metastasis.^39^ These results revealed the major activities of LINC01419 in the nucleus. However, our study revealed a novel role of LINC01419 by uncovering its glucose‐induced upregulation and cytoplasmic function as a modulator of glucose metabolism in HCC. Specifically, we are the first to identify LINC01419 as a glucose‐dependent upregulated lncRNA that binds to YBX1 in the cytoplasm to increase the mRNA stability of the metabolic enzyme PDK1, promoting reprogramming of the glycolytic pathway. Our findings underscore the significant regulatory function of LINC01419 in HCC metabolism, providing additional insight into the mechanism and therapeutic value of LINC01419 in HCC (Figure 7).

Cancer cells require substantial energy to support their rapid growth and persistence, thus metabolic reprogramming is crucial, and high glucose levels provide a readily available and abundant energy source for cancer cells.^58^, ^59^ We found that LINC01419 was upregulated in reaction to high glucose levels and increased the expression of PDK1, resulting in the reprogramming of glucose metabolism and redirecting glucose metabolism to lactate production, thus providing the energy necessary for the growth and viability of cancer cells. PDK1 is recognized for its role in inhibiting the activity of PDH, resulting in greater conversion of pyruvate towards lactate instead of its entry to the tricarboxylic acid cycle, thereby enhancing glycolysis.^60^ Enhanced glycolysis and the accumulation of lactate are frequently observed in different types of cancer. Contrary to being viewed as a metabolic byproduct, lactate can serve as a valuable nutritional substrate within the tumour microenvironment, thereby promoting tumour angiogenesis and metastasis.^61^ Targeting metabolic pathways that lead to lactate production, transport, and utilization may be a strategy for inhibiting tumour growth and metastasis.

YBX1 belongs to the RBP family and plays crucial roles in mRNP complex formation, microRNA maturation, mRNA stability and translation regulation.^62^ Here we identified the regulatory role of LINC01419 and YBX1 in the glycolytic pathway in HCC and demonstrated that LINC01419 improved the mRNA stability of PDK1 by promoting the binding of the 3′UTR of PDK1 to YBX1. Previous research has indicated that FTO promotes PDK1 expression by preventing the YTHDF2‐induced degradation of PDK1 mRNA via an m6A‐dependent mechanism, thereby facilitating the proliferation and migration of clear cell renal cell carcinoma.^63^ Cytoplasmic YBX1 has been recognized as an m5C reader that recognizes m5C‐modified mRNAs via the indole ring of W65 within its cold‐shock domain and maintaining the stabilization of its target mRNA by recruiting the ELAVL1 protein.^43^ For example, YBX1 stabilizes APOL1 mRNA by identifying and binding to the m5C site located in its 3′UTR.^64^ We also explored whether the stabilization of PDK1 mRNA by LINC01419 through YBX1 was dependent on m5C modification. By referring to m5C‐RIP‐seq data for Huh7 and HepG2 cells from the GSE221793 dataset,^65^ we found that neither LINC01419 nor PDK1 mRNA was modified by m5C in HCC. With respect to the mechanisms by which LINC01419 stabilizes PDK1 mRNA expression through YBX1 binding, the interaction between LINC01419 and YBX1 may induce a conformational change in YBX1, increasing its affinity for PDK1 mRNA and other target RNAs.^66^ On the other hand, LINC01419 may promote greater binding of PDK1 mRNA to YBX1, reduce its interaction with mRNA decay proteins or impair miRNA‐directed mRNA decay, thereby stabilizing PDK1 mRNA.^67^ The exact mechanism needs to be further elucidated and explored in subsequent studies.

YY1 was reported to be upregulated and to translocate to the nucleus in human proximal tubular cells in a glucose‐dependent manner through the mTORC1/p70S6K pathway.^68^ Glucose significantly enhances O‐linked N‐acetylglucosaminylation of YY1. Once glycosylated, YY1 loses its binding affinity for the retinoblastoma (Rb) protein enabling it to bind to DNA unimpeded, thus promoting the transcription of its target genes.^69^ In our study, YY1 was induced and translocated into the nucleus under high‐glucose conditions, where it subsequently bound to the hypomethylated LINC01419 promoter region with increased H3K27ac occupancy, thereby resulting in the specific transcriptional activation of LINC01419 in HCC cells.

Recent studies have focused on targeting cancer metabolism in vivo via siRNA/ASOs, which have yielded promising outcomes. In vivo administration of a DLGAP1‐AS2‐targeting ASO in patient‐derived xenograft (PDX) models of squamous cell carcinoma (SCC) sensitized SCC samples to cisplatin treatment.^70^ In an orthotopic glioblastoma (GBM) xenograft model, the injection of cholesterol‐modified CamK‐A siRNA targeting LINC00978 efficiently suppressed GBM cell growth and significantly extended the lifespan of mice.^71^ In this study, we observed that LINC01419 was significantly upregulated in HCC tissues but almost absent in normal liver tissues, highlighting its promise as a valuable target for developing therapeutic strategies in HCC. GalNAc conjugation of siRNAs/ASOs is considered an ideal strategy for active liver‐targeted delivery, as this modification significantly reduces the toxicity of subcutaneously injected siRNAs and ASOs.^72^ Dual‐specificity therapy can be achieved via hepatic targeting of GalNAc and specific expression of LINC01419 in HCC. Here, we found that the administration of GalNAc‐siLINC01419 notably inhibited the growth of orthotopic HCC xenografts with significant downregulation of the target gene PDK1. Additional studies are required to evaluate the efficacy of GalNAc‐siLINC01419 in HCC PDX models and organoids. Targeted lncRNA therapy can interfere with metabolic pathways to achieve therapeutic effects. Given the ubiquitous expression of metabolic enzymes across tissues, developing drugs that target these enzymes poses a formidable challenge.^8^, ^73^, ^74^ Conversely, the tissue‐restricted expression pattern of LINC01419 presents a unique opportunity for selective intervention, making it an exceptional therapeutic target. Notably, the application of GalNAc‐conjugated siRNAs targeting lncRNAs for HCC treatment remains scarce. Our findings present a novel target and an innovative therapeutic strategy for patients with advanced HCC.

In conclusion, LINC01419 is specifically upregulated by the transcription factor YY1 with increasing glucose concentrations and participates in the progression of HCC by regulating glucose metabolism. LINC01419 interacts with YBX1 in the cytoplasm and enhances the mRNA stability of the glycolytic enzyme PDK1. The newly identified LINC01419/YBX1‐PDK1 axis constitutes a valuable target, and hepatic‐specific delivery of GalNAc‐siLINC01419 presents a potential therapeutic approach for HCC. These findings suggest that LINC01419 may serve as a key molecular target involved in glucose metabolic reprogramming in HCC cells to achieve precise treatment for HCC patients.

## AUTHOR CONTRIBUTIONS

Xianghuo He and Yanfang Liu conceived and designed the study. Yanfang Liu, Junjiao Song, Wenying Qiu, and Yizhe Liu performed the experiments. Yanfang Liu, Junjiao Song, Qili Shi, Bing Chen, and Shenglin Huang processed the data. Yanfang Liu and Xianghuo He wrote and revised the manuscript. All authors read and approved the final manuscript.

## CONFLICT OF INTEREST STATEMENT

The authors declare no conflict of interest.

## ETHICS STATEMENT

All animal experiments were approved and conducted in accordance with the institutional guidelines of the Institutional Animal Care and Use Committee of Fudan University Shanghai Cancer Center (permission number: FUSCC‐IACUC‐2022282), Shanghai, China.

## Supporting information



Supporting Information

Supporting Information

Supporting Information

Supporting Information

## Data Availability

Data supporting the findings of the current study are listed in NCBI Gene Expression Omnibus (accession no: GSE234898).
