# Supplementary material for: Glucose‐induced LINC01419 reprograms the glycolytic pathway by recruiting YBX1 to enhance PDK1 mRNA stability in hepatocellular carcinoma
Source: Clin Transl Med. 2024 Dec 3;14(12):e70122. doi: 10.1002/ctm2.70122 (PMC11613097; doi:10.1002/ctm2.70122)
Supplement: Supplementary file 1 — Supporting Information [file CTM2-14-e70122-s004.docx]

**Supplementary Information for**

**Glucose-Induced LINC01419 Reprograms the Glycolytic Pathway by Recruiting YBX1 to Enhance PDK1 mRNA Stability in Hepatocellular Carcinoma**

Yanfang Liu^1,#^, Junjiao Song^1,#^, Qili Shi^1^, Bing Chen^1^, Wenying Qiu^1^, Yizhe Liu^1^, Shenglin Huang^1,2^, Xianghuo He^1,2,3^.

^1^Fudan University Shanghai Cancer Center and Institutes of Biomedical Sciences, Department of Oncology, Shanghai Medical College, Fudan University, Shanghai 200032, China

^2^Key Laboratory of Breast Cancer in Shanghai, Fudan University Shanghai Cancer Center, Fudan University, Shanghai 200032, China.

^3^Collaborative Innovation Center for Cancer Personalized Medicine, Nanjing University, Nanjing 211166, China

^#^These authors contributed equally to this work.

**Corresponding Author:**

Xianghuo He, Email: [xhhe@fudan.edu.cn](mailto:xhhe@fudan.edu.cn), Fudan University Shanghai Cancer Center and Institutes of Biomedical Sciences; Department of Oncology, Shanghai Medical College, Fudan University, 302 Rm., 7# Bldg., 27 270 Dong An Road, Shanghai 200032, China. Tel: 86-21-34777329; Fax: 86-21-64172585.

**Supplementary Materials and Methods**

**Cell culture and reagents**

Huh7 cells were obtained from the Japanese Collection of Research Bioresources (JCRB, Tokyo, Japan). HEK-293T cells were obtained from the American Type Culture Collection (ATCC, Manassas, Virginia, USA). The PLC, SK-Hep1 and HepG2 cell lines were purchased from the Shanghai Cell Bank Type Culture Collection Committee (Shanghai, China). All the cell lines were cultured in high-glucose (4.5 g/L, 25 mM) DMEM supplemented with 10% fetal bovine serum (FBS) at 37 °C in 5% CO_2_. For low-glucose culture, the glucose concentration was 1 g/L (5 mM). D-Glucopyranose and 5-AzaC were purchased from Sigma‒Aldrich.

**RNA pull-down**

Biotin-labeled RNAs (LINC01419-sense/anti-sense; LINC01419-A/B/C; PDK1-3’UTR) were transcribed in vitro with the Biotin RNA Labeling Mix (Roche, Basel, Switzerland) and T7 RNA polymerase (New England Biolabs, MA, USA) according to the manufacturer’s instructions. Biotin-labeled RNAs were incubated with cell lysates in tubes at 4 °C for 4 h, and 30 μl of streptavidin magnetic beads (Invitrogen, CA, USA) was added to the solution and incubated at 4 °C for 1 h. After 6 thorough washes with NT2 buffer (300 mM NaCl for the last 3 washes), proteins binding to the biotin-labeled RNA on the magnetic beads were collected by boiling with 1× SDS loading buffer at 100 °C for 10 min and were then analyzed by western blot. The primers used in the RNA pull-down assays are listed in **Supplementary Table S3A**.

**RIP assay**

Plates containing cells were placed on ice for UV crosslinking, collected into tubes and lysed with RIP lysis buffer. Then, the cell lysates were incubated with antibodies (anti-YBX1 or anti-CCAR1) at 4 °C for 4 h, and 30 μl of protein G magnetic beads was added for further incubation at 4 °C for 2 h. Beads conjugated to protein-RNA complexes were washed with NT2 buffer six times, after which 1/10 of the volume of beads was used for protein extraction to determine the efficiency of immunoprecipitation, and RNA in complexes was eluted from the remaining beads with TRIzol reagent (Invitrogen, CA, USA). Protein-interacting RNA was extracted for sequencing or use in equal volumes for reverse transcription for qPCR analysis.

**In vitro RNA-protein binding assays**

In vitro protein expression and purification: The YBX1 gene was expressed using the pET-45b vector with a lacl-controlled T7 promoter. Plasmids were transferred into BL21 competent cells, and protein expression was induced by isopropyl β-D-1-thiogalactopyranoside (IPTG). Ni IDA Beads 6FF (Smart-Lifesciences, Jiangsu, China) were used for subsequent purification.

Biotinylated LINC01419/ PDK1-3’UTR (0.1 μg) was incubated with 0.5 μg, 2 μg, or 4 μg YBX1 protein at room temperature (RT). One hour later, 30 μl of streptavidin magnetic beads was added to the system for incubation at RT for 30 min. The beads were thoroughly washed with NT2 buffer 6 times (300 mM NaCl for the last 3 washes) and then incubated with 1× SDS loading buffer at 100 °C for 10 min. The retrieved proteins were analyzed by western blot.

**Glucose uptake, lactate production and ATP measurement**

For metabolite measurements, the cell culture medium was replaced with phenol red-free DMEM/F12 15 h before the tests. The glucose content in the medium was measured with a Glucose Assay Kit with O-Toluidine (Beyotime, Shanghai, China, Cat. No. S0201S). Lactate production was measured by a CheKine Micro Lactate Assay Kit (Abbkine, California, USA, Cat. No. KTB1100). The intracellular ATP levels in cells were measured by an ATP Assay Kit (Beyotime, Shanghai, China, Cat. No. S0026). All operations were carried out according to the manufacturer's instructions.

**RNA isolation and quantitative PCR (qPCR) analyses**

RNA was extracted with TRIzol reagent (Invitrogen, CA, USA). qPCR analysis was performed using the Evo M-MLV RT Master Mix and SYBR Green Pro Taq HS (Accurate Biology, Hunan, China). Primers used for qPCR are listed in **Supplementary Table S3B**.

**Subcellular fractionation**

A total of 1×10^6^ cells were lysed with subcellular fractionation lysis buffer (20 mM Tris-HCl (pH 7.5), 2 mM MgCl_2_, 10 mM NaCl, 0.1% NP40) on ice for 5~10 mins. Following centrifugation at 12000 g and 4 °C for 1 min, the supernatant was collected as the cytoplasmic fraction, and the nuclear pellet was rinsed twice with precooled PBS. RNA was extracted from the cytoplasmic and nuclear fractions using TRIzol reagent and analyzed by qPCR. Proteins were extracted with 1× SDS loading buffer and analyzed by western blot.

**RNA FISH**

RNA FISH assays were performed using the Fluorescence In Situ Hybridization Kit (RiboBio) according to the manufacturer’s instructions. The LINC01419 probes were synthesized by RiboBio (Guangzhou, China).

**3’ RACE and 5’ RACE**

3’ RACE and 5’ RACE analyses were performed with the SMARTer RACE 5’/3’ Kit (TaKaRa, Shiga, Japan) according to the manufacturer's instructions. The gene-specific PCR primers used for RACE analyses are listed in **Supplementary Table S3C**.

**Cell transfection**

siRNA-mediated gene knockdown was performed using RNAiMAX Transfection Reagent (Invitrogen, CA, USA) with siRNA at a final concentration of 25 nM. The cells were harvested for cell functional experiments or for RNA and protein analysis 48 h after transfection. The sequences of the siRNAs used in this study are listed in **Supplementary Table S3D**.

Plasmids were transfected into cells using Hieff Trans Liposomal Transfection Reagent (Yeasen, Shanghai, China) according to the manufacturer’s instructions.

**Plasmid construction, lentivirus packaging and infection**

The full length LINC01419 sequence, confirmed by RACE, and the truncated LINC01419 (LINC01419-∆C) was amplified and inserted into the pCDH-Puro lentiviral vector. The coding sequences (CDSs) of YBX1 and PDK1 were also amplified and inserted into the pCDH-Puro lentiviral vector. The full-length and truncated fragments of YBX1 were subcloned into the pCMV-N-Flag vector. The LINC01419-shRNA/YBX1-shRNA/PDK1-shRNA sequences were inserted into the LentiGuide-Puro lentiviral vector. The 3’UTR of PDK1 was inserted into psi-CHECK-2 vector. The promoter region of LINC01419 was inserted into pGL3.basic vector. The primer sequences containing the homologous sequences with the vector for plasmid construction are listed in **Supplementary Table S3E**.

For lentivirus packaging and infection, the lentiviral vector was cotransfected with the pMD2G envelope plasmid and pAX2 packaging plasmids into HEK-293T cells. Forty-eight hours later, the viral supernatant was collected by filtration and used to infect cells in the presence of polybrene at a final concentration of 6 μg/mL.

**Cell growth and colony formation assays**

For cell growth assays, 1000 or 1500 cells were plated in triplicate into 96-well plates, and 10 μl of CCK-8 reagent was added to the medium on days 1, 3, and 5. After 2 h of incubation, the OD450 of each well was measured by a microplate reader.

For colony formation assays, 1000, 1500, or 3000 cells were plated in triplicate wells of six-well plates and were then cultured at 37 °C in 5% CO_2_ for 8~14 days. The colonies were stained with 0.1% crystal violet in methanol, and the colonies in each well were counted.

**Transwell assays**

A total of 5×10^4^ Huh7/ 5×10^4^ PLC/ 5×10^4^ SK-Hep1/ 1×10^5^ HepG2 cells were resuspended in serum-free medium were seeded into transwell chambers with medium supplemented with 10% FBS in the compartments below the chambers. Then, 16 ~ 48 hours after incubation, the cells in the chambers were fixed and stained with 0.1% crystal violet in methanol. For invasion assays, the upper surface of the membrane in the chamber was precoated with Matrigel (BD, Bergen, New Jersey, USA).

**RNA sequencing**

Total RNA was extracted with TRIzol reagent (Invitrogen, CA, USA). For screening of the candidate lncRNAs, the RiboMinus Eukaryote Kit (QIAGEN, CA, USA) was used to remove rRNAs, and the NEBNext Ultra Directional RNA Library Prep Kit (New England Biolabs, MA, USA) was used to build the RNA-seq library. Transcript expression was analyzed using StringTie and quantified by fragments per kilobase of exon per million fragments mapped (FPKM) values. For other analyses, the mRNA-seq library was constructed using a VAHTS Stranded mRNA-seq Library Prep Kit (Vazyme Biotech, Jiangsu, China). Gene expression was analyzed using Hisat2 and quantified by FPKM values. GO-BP analysis was conducted via DAVID Bioinformatics Resources (https://david.ncifcrf.gov/), the fold changes in the FPKM values of all genes were calculated and compared between the knockdown group and negative control group, and the list of genes with a fold change of less than 0.67 was imported into the DAVID Functional Annotation tool for analysis.

**Western blot analysis**

Cellular proteins were extracted with RIPA lysis buffer (Beyotime, Shanghai, China) containing protease inhibitors (Bimake, Houston, Texas, USA) and incubated with SDS loading buffer at 100 °C for 10 mins. The samples were subjected to SDS‒PAGE, and the separated proteins were transferred to a nitrocellulose membrane (GE, Boston, Massachusetts, USA). The membrane was sequentially blocked with 5% nonfat milk (Sangon, Shanghai, China) at RT for 1 h, incubated with primary antibodies at RT for 2 h, and incubated with secondary antibodies at RT for 1 h. After washing with TBST (TBS with Tween-20), the membrane was incubated with the appropriate components of an Omni-ECL Femto Light Chemiluminescence Kit (Epizyme, Shanghai, China) and imaged by a chemiluminescence imaging system (Tanon, Shanghai, China).

**Dual-luciferase reporter assay**

The promoter of LINC01419 was inserted into the firefly luciferase reporter vector pGL3.basic (Promega, Wisconsin, USA). Cells were transfected with the plasmids alone or in combination with siRNAs using Lipofectamine 2000 Transfection Reagent (Invitrogen, CA, USA). The pRL-TK vector (Promega, Wisconsin, USA) was cotransfected as an internal control. Forty-eight hours after transfection, luciferase activity was measured using the Dual-Luciferase Reporter Assay System (Promega, Wisconsin, USA, Cat.No. E1910).

**Chromatin immunoprecipitation (ChIP)**

1 × 10^7^ cells were subjected to crosslinking with 1% formaldehyde at RT for 10 min, and 150 mM glycine was then added for termination of crosslinking. Then, the cells were washed with PBS and collected into tubes. Chromatin was digested into DNA fragments using Micrococcal Nuclease (Cell Signaling Technology, Danvers, Massachusetts, USA). Protein-DNA complexes were extracted and resuspended in SimpleChIP Chromatin IP buffers (Cell Signaling Technology, Danvers, Massachusetts, USA). The complexes were incubated with antibody-conjugated (anti- mouse IgG, anti- rabbit IgG, anti-YY1, anti-H3K27ac or anti-RNA pol II) protein G magnetic beads overnight at 4°C and washed thoroughly. Finally, crosslinking was reversed for 2 h at 65°C, and DNA was purified using MinElute Spin Columns (QIAGEN, Hilden, Germany) for qPCR analysis. The primers used in the ChIP‒qPCR assay are listed in **Supplementary Table S3F**.

**Immunofluorescence staining**

Cells were fixed with 4% paraformaldehyde for 15 mins and then washed with PBS. After incubation with immunofluorescence blocking solution (Beyotime, Shanghai, China) at RT for 1 h, the samples were incubated with an anti-YY1 antibody (Proteintech, Wuhan, China) at 4 °C overnight prior to incubation with a secondary antibody (Thermo Fisher Scientific, Waltham, MA, USA) at RT for 1 h. The slides were sealed with ProLong Gold Antifade Mountant with DNA Stain DAPI (Invitrogen, CA, USA) for observation by laser confocal microscopy (Leica, Heerbrugg, Switzerland).**Supplementary Figures**

**Supplementary Fig. S1**

**
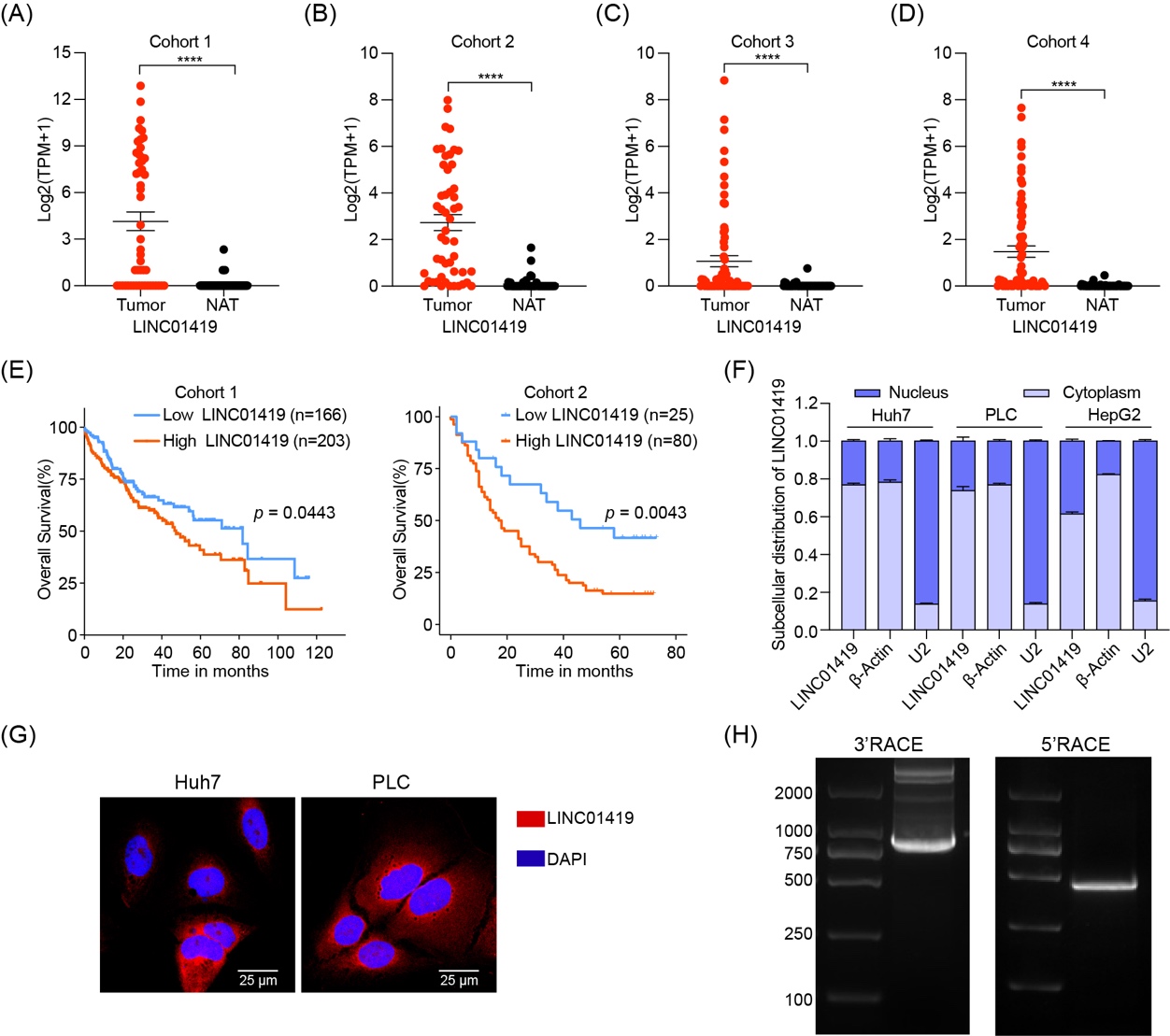
**

**Figure S1 The characteristics of LINC01419.**

**(A-D)** Relative expression of LINC01419 in HCC tissues and normal tissues adjacent to the tumors (NATs) in cohort 1 (369 HCC cases from TCGA) **(A)**, cohort 2 (105 HCC cases from the GepLiver project) **(B)**, cohort 3 (50 HCC cases from GSE77314) **(C)** and cohort 4 (70 HCC cases from GSE144269) **(D)**. **(E)** Kaplan–Meier survival analysis to determine the correlation between the LINC01419 expression level and overall survival in HCC patients in cohort 1 (left) and cohort 2 (right). **(F)** Subcellular fractionation followed by qPCR was performed to determine the relative subcellular distribution of LINC01419 in HCC cells (Huh7, PLC and HepG2). U2 served as a nuclear marker, and β-actin served as a cytoplasmic marker. **(G)** RNA FISH was performed to detect the cellular localization of LINC01419 in Huh7 and PLC cells. Scale bar, 25 μm. **(H)** Identification of the full-length LINC01419 sequence in Huh7 cells by 3’ RACE and 5’ RACE. The data are presented as the means ± SDs. ****P < 0.0001.

**Supplementary Fig. S2**


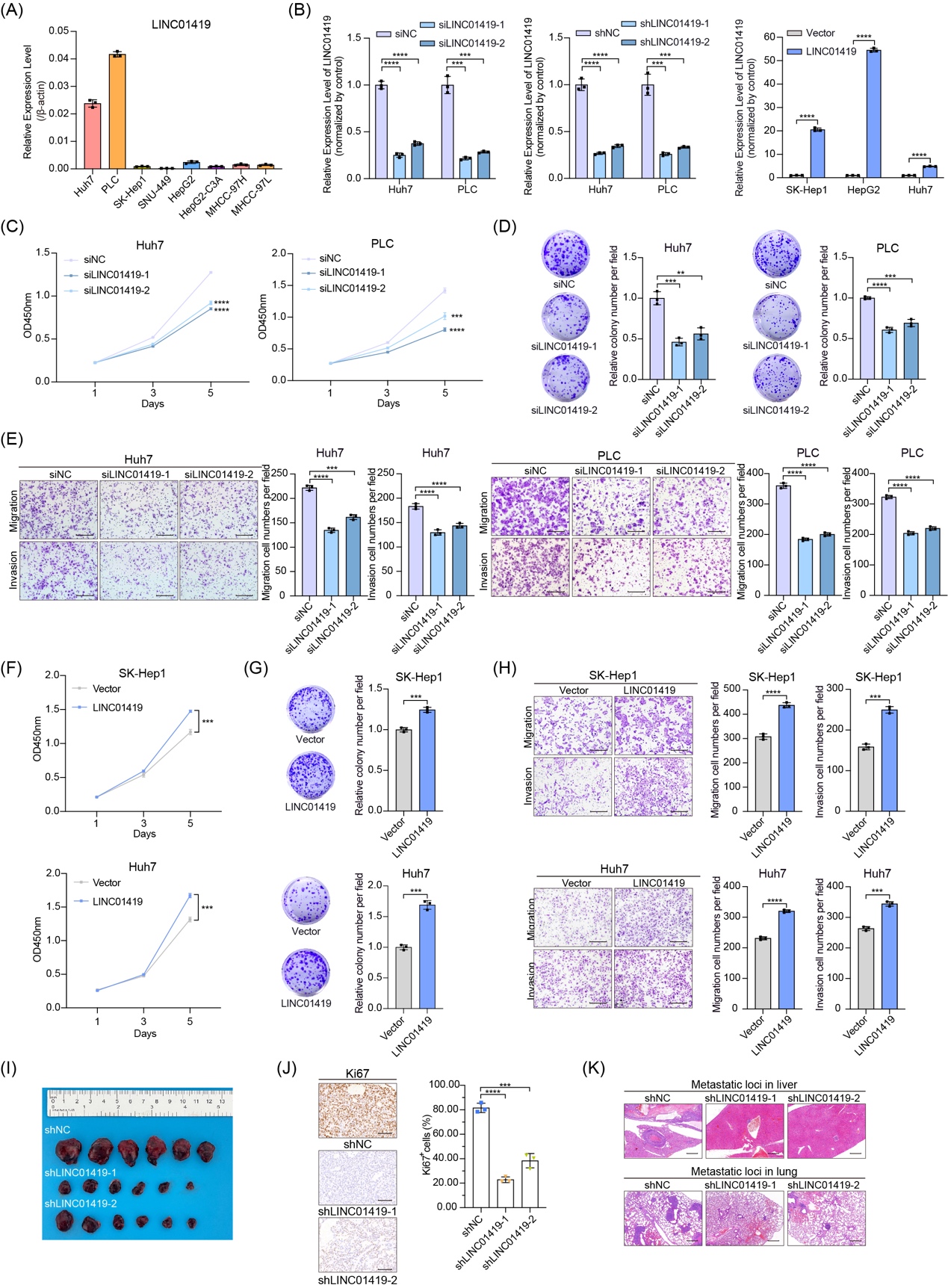


**Figure S2 LINC01419 promotes HCC cell growth and metastasis in vitro and in vivo.**

**(A)** Relative expression levels of LINC01419 in HCC cell lines was determined by qPCR, β-actin was used as the reference. **(B)** Determination of the knockdown or overexpression efficiency of LINC01419 by qPCR analysis (left: knockdown by siRNA; right: stable overexpression). **(C)** CCK-8 assays of Huh7 and PLC cells following knockdown of LINC01419 by siRNA (NC as the control). **(D)** Colony formation assays of Huh7 and PLC cells following knockdown of LINC01419 by siRNA (NC as the control). **(E)** Migration and invasion assays of Huh7 and PLC cells following knockdown of LINC01419 by siRNA (NC as the control). Scale bar, 200 μm. **(F-H)** CCK-8 **(F)**, colony formation **(G)**, migration and invasion **(H)** assays of SK-Hep1 and Huh7 cells following stable overexpression of LINC01419 (Vector as the control). Scale bar, 200 μm. **(I)** Images of subcutaneous tumor tissues from individual groups (right). **(J)** Representative images of Ki67 IHC staining in mouse subcutaneous tumors formed by Huh7 cells following NC or knockdown of LINC01419 by shRNA (left) and statistical results of ki67-positive cells (right). Scale bar, 100 μm. **(K)** Representative images of H&E-stained liver and lung tissues from each group of orthotopic xenograft model mice. Scale bar, 500 μm. The data are presented as the means ± SDs. **P < 0.01, ***P < 0.001, ****P < 0.0001.

**Supplementary Fig. S3**


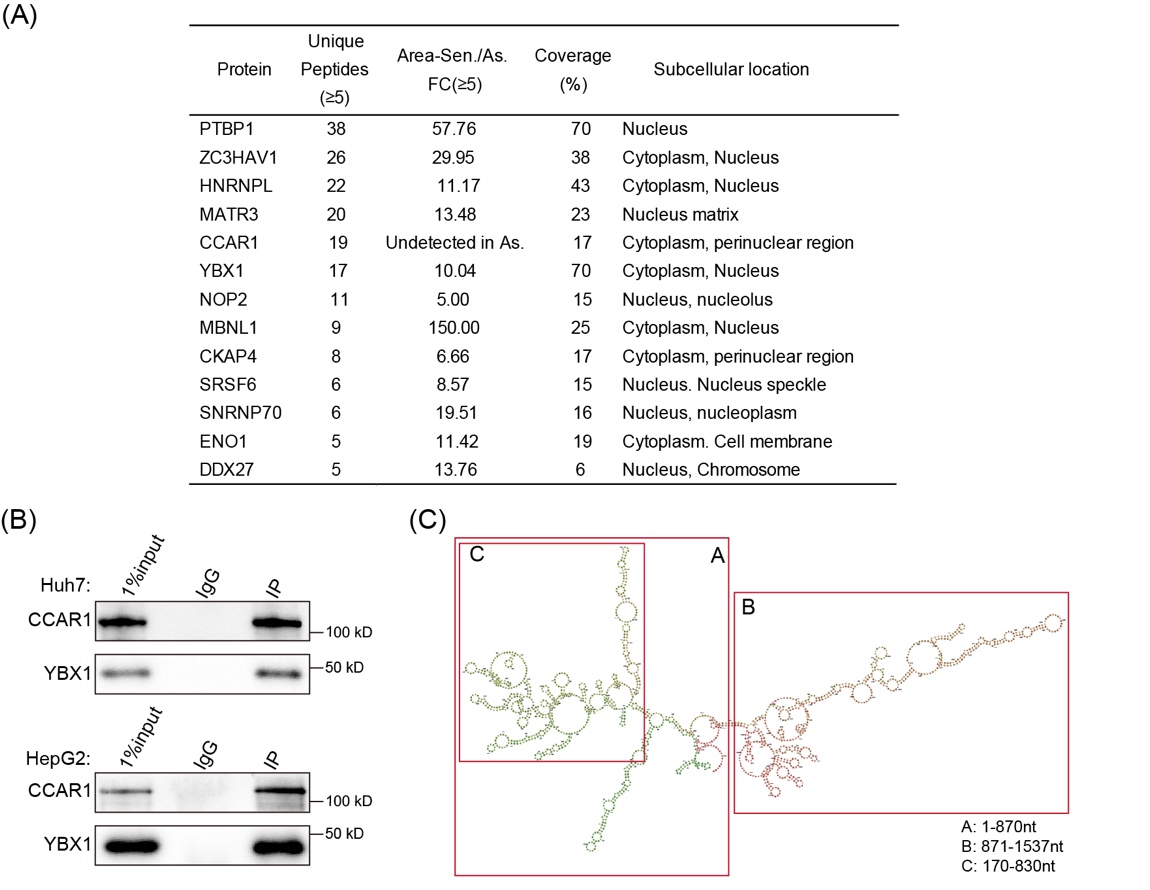


**Figure S3 LINC01419 directly interacts with YBX1 in HCC cells.**

**(A)** Filtered list of RNA pull-down proteins detected of by mass spectrometry. **(B)** The immunoprecipitation efficiency of CCAR1 and YBX1 in Huh7 and HepG2 cells was evaluated by western blot analysis. **(C)** Secondary structure of LINC01419 predicted with the RNAfold web server (<http://rna.tbi.univie.ac.at/>).

**Supplementary Fig. S4**

**
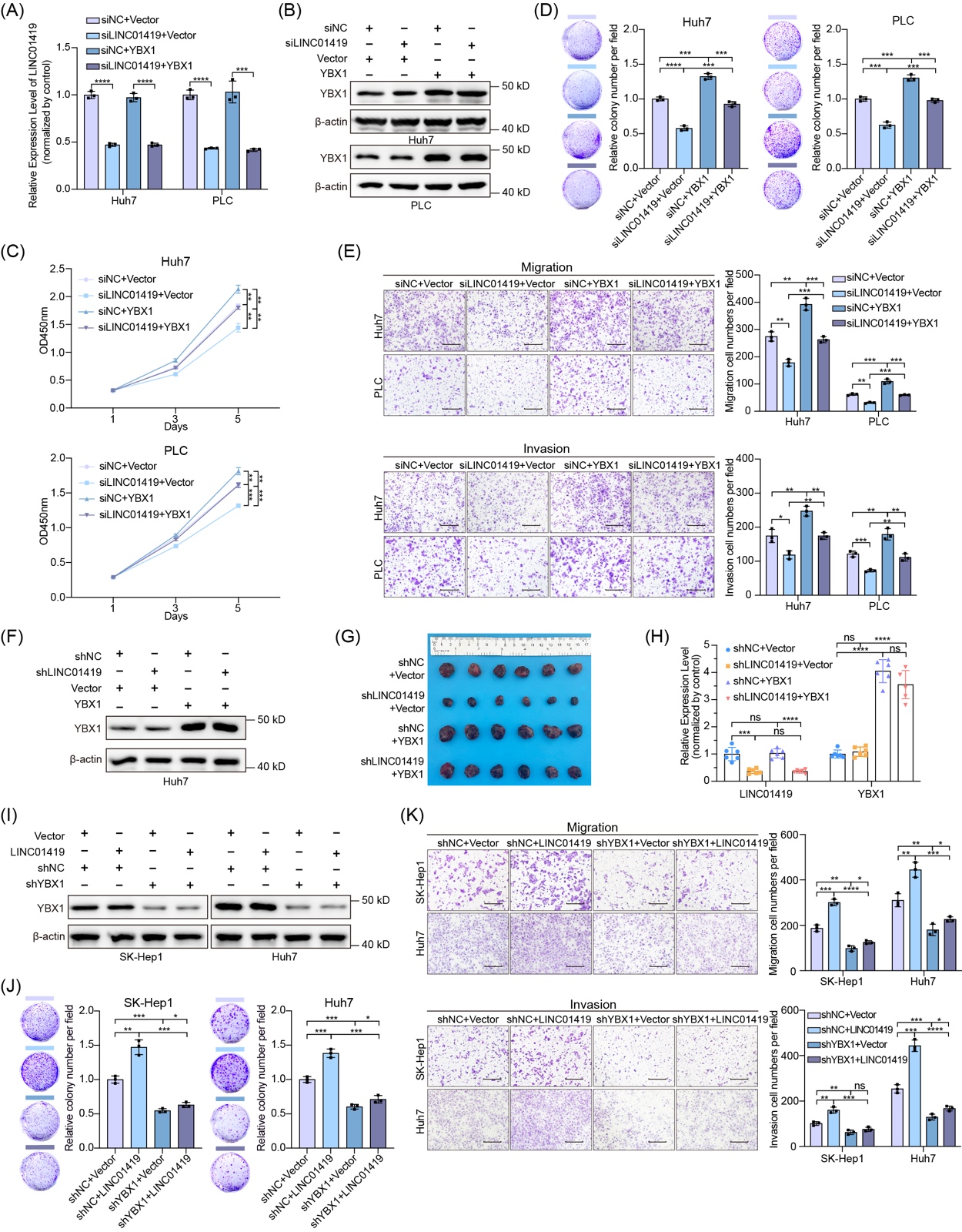
**

**Figure S4 YBX1 acts as the downstream mediator of LINC01419 in HCC cells.**

**(A)** The knockdown efficiency of LINC01419 by siRNA in Huh7 and PLC cells was determined by qPCR. **(B)** The overexpression efficiency of YBX1 in Huh7 and PLC cells was determined by western blot. **(C)** CCK-8 assays of Huh7 and PLC cells with knockdown of LINC01419 by siRNA and overexpression of YBX1. **(D)** Colony formation assays of Huh7 and PLC cells with knockdown of LINC01419 by siRNA and overexpression of YBX1. **(E)** Migration (top) and invasion (bottom) assays of Huh7 and PLC cells with the indicated treatments. Scale bar, 200 μm. **(F)** The overexpression efficiency of YBX1 protein in Huh7 cells following knockdown of LINC01419 by shRNA was detected by western blot. **(G)** Mice were injected subcutaneously with 2×10^6^ Huh7 cells following knockdown of LINC01419 and overexpression of YBX1 (shNC and vector as the control), tumor volume was measured every 2 to 3 days after tumor formation. Image of subcutaneous tumor tissues from individual groups. **(H)** The relative expression of LINC01419 and YBX1 was detected in tumor tissues of each group by qPCR (n=6). **(I)** The knockdown efficiency of YBX1 protein in SK-Hep1 and Huh7 cells following overexpression of LINC01419 was detected by western blot. **(J)** Colony formation assays of SK-Hep1 and Huh7 cells following overexpression of LINC01419 and knockdown of YBX1 by shRNA. **(K)** Migration (top) and invasion (bottom) assays of Huh7 and PLC cells with the indicated treatments. Scale bar, 200 μm. The data are presented as the means ± SDs. *P < 0.05, **P < 0.01, ***P < 0.001, ****P < 0.0001; differences with P > 0.05 were considered nonsignificant (ns).**Supplementary Fig. S5**


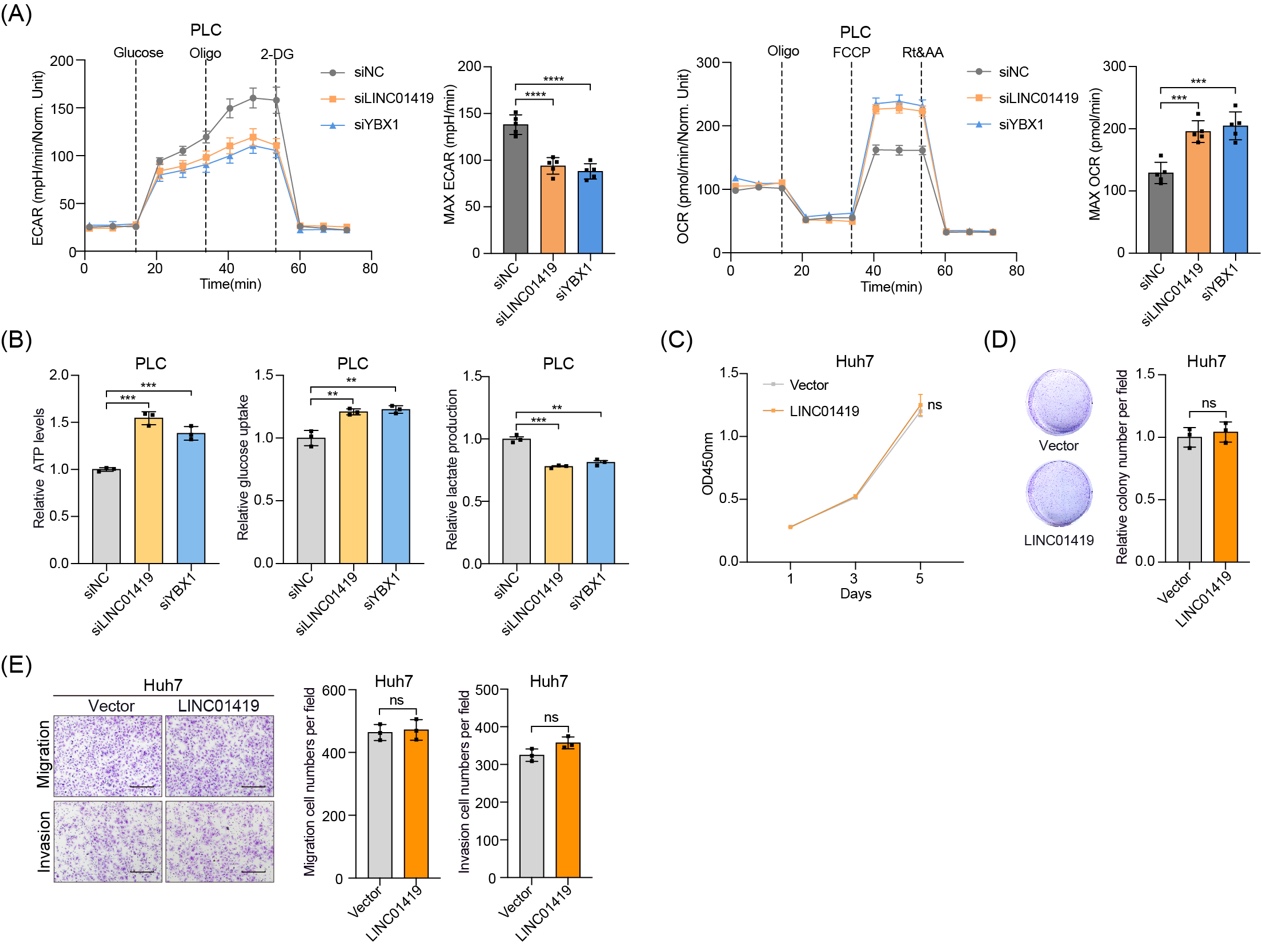


**Figure S5 LINC01419 and YBX1 reprogram glucose metabolism.**

**(A)** Extracellular acid ratio (ECAR) and Oxygen consumption ratio (OCR) in PLC cells following knockdown of LINC01419 or YBX1 by siRNA by Seahorse Metabolic Analyzer. The MAX ECAR/OCR was analyzed on the right side. **(B)** Relative ATP levels, glucose uptake and lactate production in PLC cells following knockdown of LINC01419 or YBX1 by siRNA. **(C-E)** CCK-8 **(C)**, colony formation **(D)**, migration and invasion **(E)** assays of Huh7 cells following stable overexpression of LINC01419 cultured in medium containing galactose instead of glucose. Scale bar, 200 μm. The data are presented as the means ± SDs. **P < 0.01, ***P < 0.001, ****P < 0.0001; differences with P > 0.05 were considered nonsignificant (ns).

**Supplementary Fig. S6**


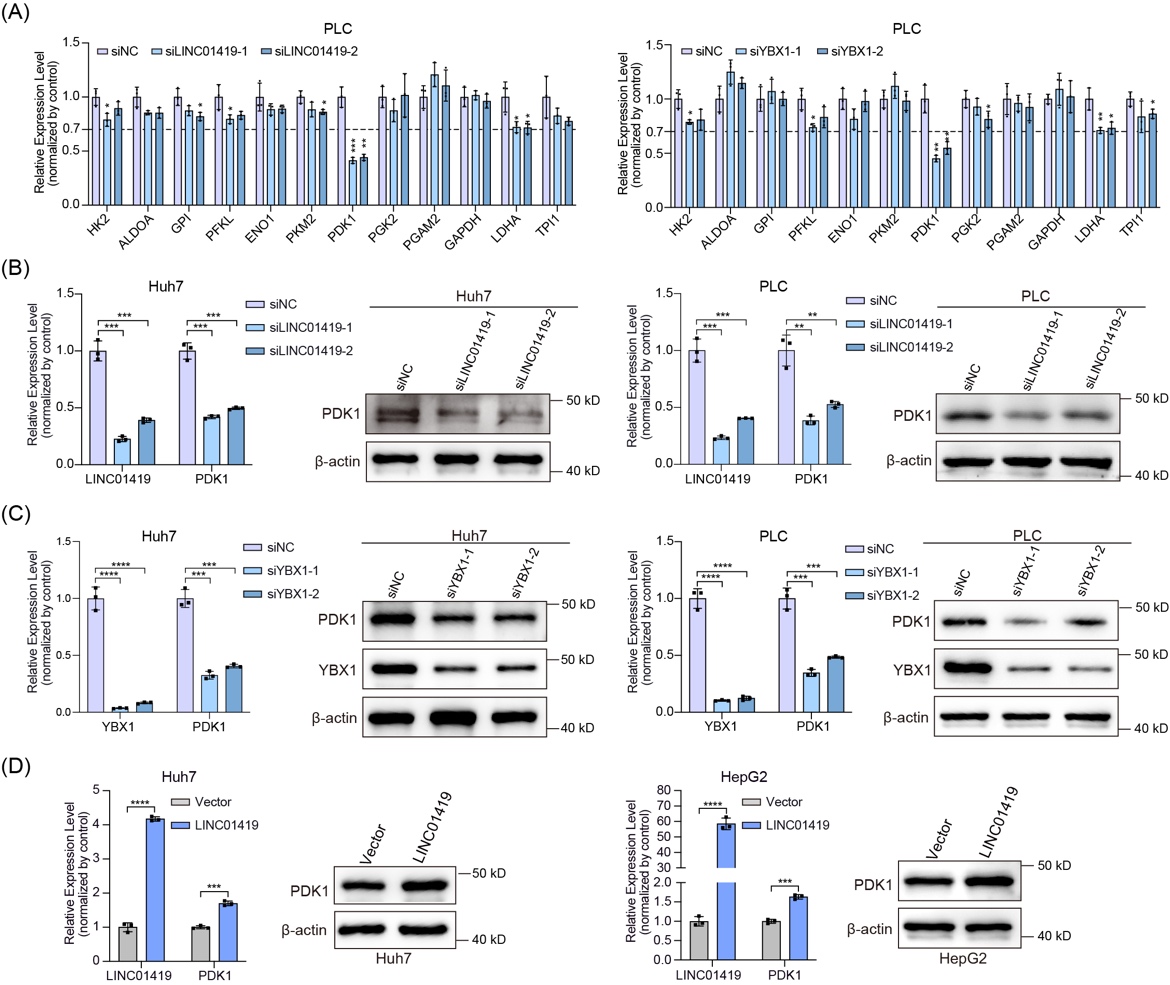


**Figure S6 LINC01419 and YBX1 reprogram glucose metabolism by increasing PDK1 expression in HCC cells.**

**(A)** Relative expression levels of glycolytic enzymes in PLC cells following knockdown of LINC01419 or YBX1, as determined by qPCR. **(B)** The expression of PDK1 in Huh7 and PLC cells following knockdown of LINC01419 was analyzed by qPCR and western blot. **(C)** The expression of PDK1 in Huh7 and PLC cells following knockdown of YBX1 was analyzed by qPCR and western blot. **(D)** The expression of PDK1 in Huh7 and HepG2 cells following stable overexpression of LINC01419 was analyzed by qPCR and western blot. The data are presented as the means ± SDs. *P < 0.05, **P < 0.01, ***P < 0.001, ****P < 0.0001.**Supplementary Fig. S7**


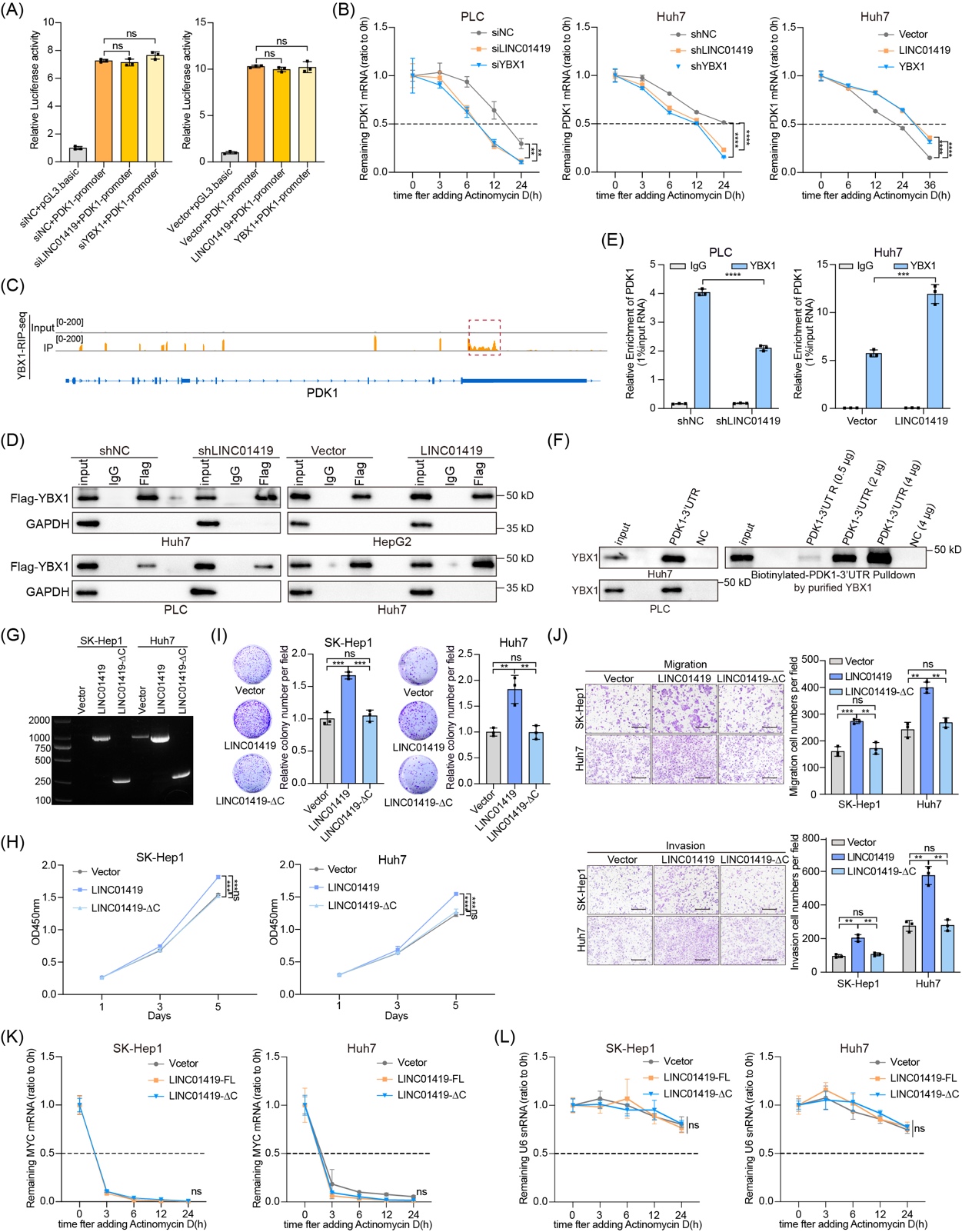


**Figure S7 LINC01419 enhances PDK1 mRNA stability via interaction with YBX1.**

**(A)** The relative luciferase activity of the PDK1 promoter was determined by a dual luciferase assay after knockdown (left) or overexpression (right) of LINC01419 or YBX1. **(B)** Relative RNA expression of PDK1 in PLC cells following knockdown of LINC01419 or YBX1 by siRNA (left), in Huh7 cells following knockdown of LINC01419 or YBX1 by shRNA (middle) and in Huh7 cells following stable overexpression of LINC01419 or YBX1 (right) at different time points after treatment with actinomycin D (5 μg/mL) was detected by qPCR. **(C)** The enrichment of YBX1 in the region of PDK1 was evaluated by YBX1-RIP-seq in Huh7 cells, and the peaks were aligned to the hg38 genome. **(D)** Western blot analysis was performed to determine the RIP efficiency of Flag-YBX1 in HCC cells. **(E)** The enrichment of YBX1 in the region of PDK1 was evaluated in PLC cells following knockdown of LINC01419 (shNC as the control) and Huh7 cells following overexpression of LINC01419 (Vector as the control) by RIP-qPCR. **(F)** The expression of YBX1 in pull-down proteins of Huh7 and PLC cellular whole proteins by biotin-labeled PDK1-3’UTR RNA (left) and the enrichment of YBX1 in the in vitro binding assay of biotin-labeled PDK1-3’UTR RNA to YBX1 (right) was detected by western blot (LINC01419-antisense as Negative Control). **(G)** The expression of full-length and truncated LINC01419 (LINC01419-∆C) in SK-Hep1 and Huh7 cells was detected by RT-PCR. **(H)** CCK-8 assays of SK-Hep1 and Huh7 cells with stable overexpression of LINC01419 and its truncation form LINC01419-∆C (Vector as the control). **(I)** Colony formation assays of SK-Hep1 and Huh7 cells with stable overexpression of LINC01419 and its truncation form LINC01419-∆C (Vector as the control). **(J)** Migration (top) and invasion (bottom) assays of SK-Hep1 and Huh7 cells with stable overexpression of LINC01419 and its truncation form LINC01419-∆C (Vector as the control). Scale bar, 200 μm. **(K, L)** Relative RNA expression of MYC **(K)** and U6 **(L)** as controls in SK-Hep1 and Huh7 cells following stable overexpression of full-length and truncated LINC01419 (LINC01419-∆C) at different time points after treatment with actinomycin D (5 μg/mL) was detected by qPCR. The data are presented as the means ± SDs. **P < 0.01, ***P < 0.001, ****P < 0.0001; differences with P > 0.05 were considered nonsignificant (ns).

**Supplementary Fig. S8**

**
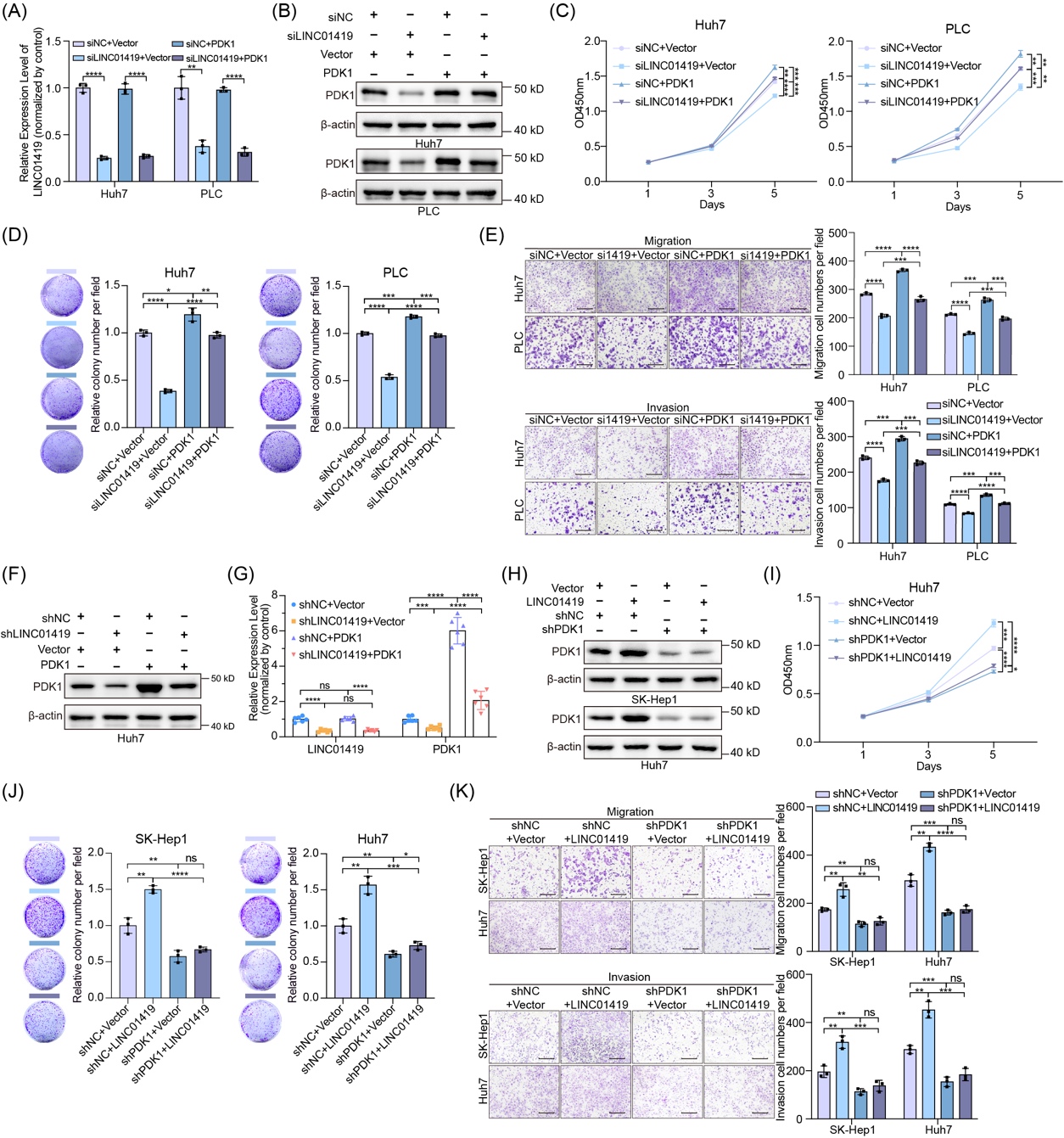
**

**Figure S8 PDK1 is a functional target of LINC01419.**

**(A)** The knockdown efficiency of LINC01419 by siRNA in Huh7 and PLC cells was determined by qPCR. **(B)** The overexpression efficiency of PDK1 in Huh7 and PLC cells was determined by western blot. **(C)** CCK-8 assays of Huh7 (left) and PLC (right) cells following knockdown of LINC01419 by siRNA and overexpression of PDK1. **(D)** Colony formation assays of Huh7 (left) and PLC (right) cells following knockdown of LINC01419 by siRNA and overexpression of PDK1, the colors represent different groups. **(E)** Migration (top) and invasion (bottom) assays of Huh7 and PLC cells with the indicated treatment. Scale bar, 200 μm. **(F)** The overexpression efficiency of PDK1 in Huh7 cells following knockdown of LINC01419 by shRNA was determined by western blot. **(G)** The relative expression of LINC01419 and PDK1 was detected in subcutaneous tumor tissues of each group by qPCR (n=6). **(H)** The knockdown efficiency of PDK1 protein in SK-Hep1 and Huh7 cells following overexpression of LINC01419 was detected by western blot. **(I)** CCK-8 assays of Huh7 cells following stable overexpression of LINC01419 and knockdown of PDK1 by shRNA (shNC and vector as the control). **(J)** Colony formation assays of SK-Hep1 and Huh7 cells following stable overexpression of LINC01419 and knockdown of PDK1 by shRNA (shNC and vector as the control). **(K)** Migration (top) and invasion (bottom) assays of SK-Hep1 and Huh7 cells following stable overexpression of LINC01419 and knockdown of PDK1 by shRNA (shNC and vector as the control). Scale bar, 200 μm. The data are presented as the means ± SDs. *P < 0.05, **P < 0.01, ***P < 0.001, ****P < 0.0001; differences with P > 0.05 were considered nonsignificant (ns).

**Supplementary Fig. S9**

**
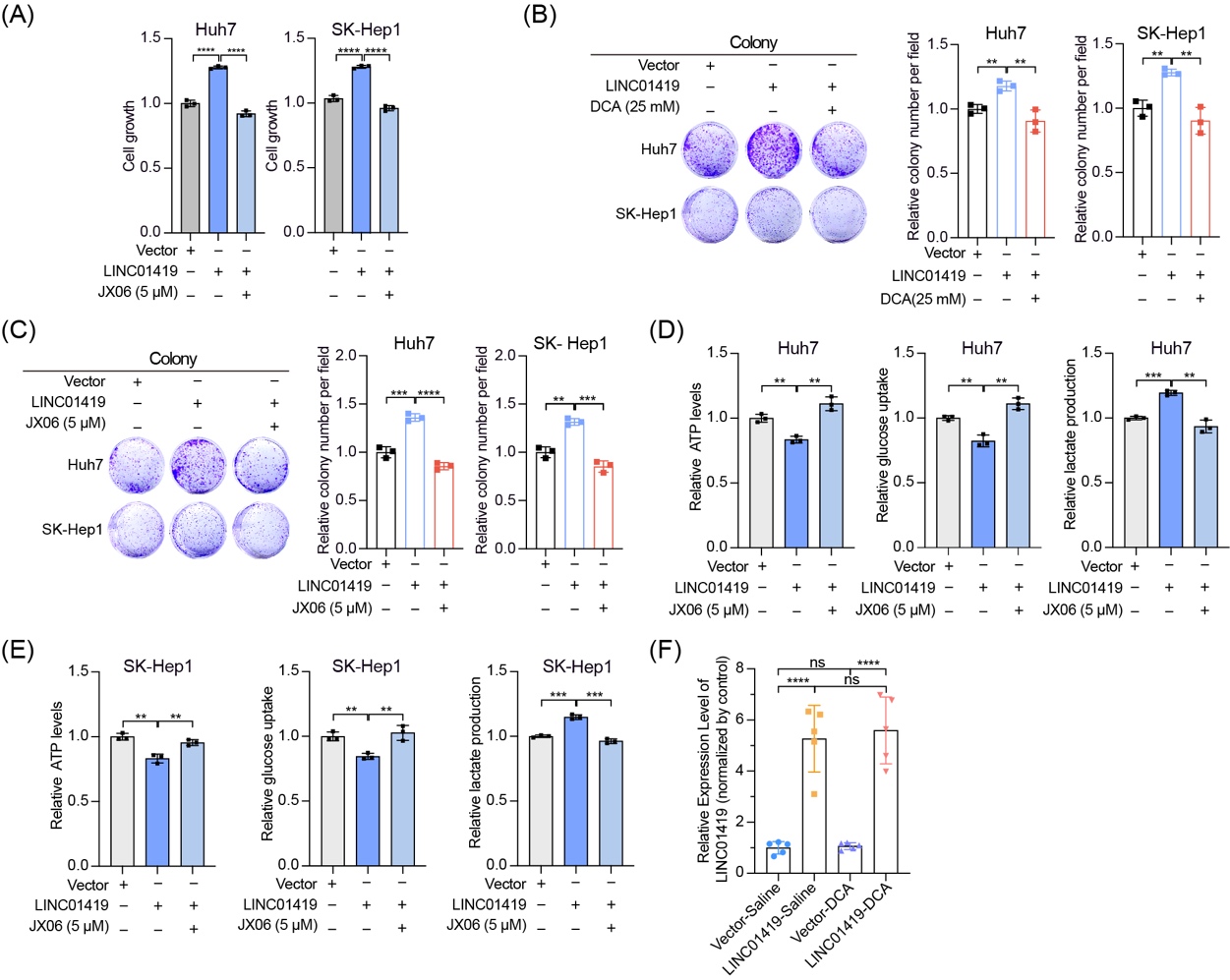
**

**Figure S9 PDK1 inhibitors DCA and JX06 significantly suppress LINC01419-induced tumor proliferation.**

**(A)** CCK-8 assays of Huh7 and SK-Hep1 cells following overexpression of LINC01419 and treatment with JX06 (5 μM) for 24 h. **(B)** Colony assays of Huh7 and SK-Hep1 cells following overexpression of LINC01419 and treatment with DCA (25 mM). **(C)** Colony assays of Huh7 and SK-Hep1 cells following overexpression of LINC01419 and treatment with JX06 (5 μM). **(D, E)** Relative ATP levels (left), glucose uptake (middle) and lactate production (right) in Huh7 **(D)** and SK-Hep1 **(E)** cells following overexpression of LINC01419 and treatment with JX06 (5 μM) for 24 h. **(F)** The relative expression of LINC01419 was detected in subcutaneous tumor tissues of each group by qPCR (n=5). The data are presented as the means ± SDs. **P < 0.01, ***P < 0.001, ****P < 0.0001; differences with P > 0.05 were considered nonsignificant (ns).

**Supplementary Fig. S10**


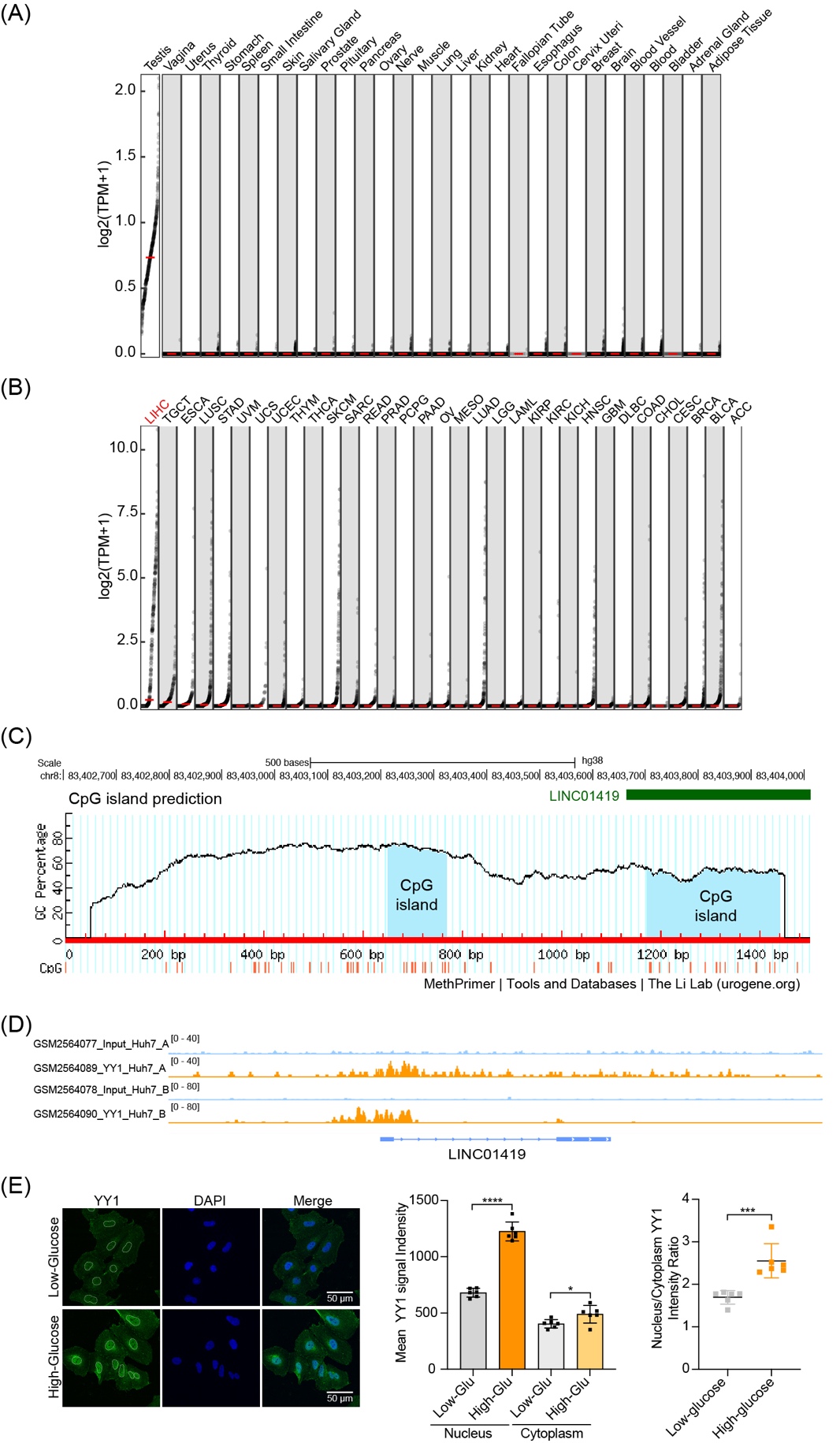


**Figure S10 LINC01419 is regulated by the transcription factor YY1.**

**(A)** The expression of LINC01419 in normal human tissues in the GTEx database. **(B)** The expression of LINC01419 across cancers in the TCGA database (ACC adrenocortical carcinoma, BLCA Bladder Urothelial Carcinoma, LAML Acute Myeloid Leukemia, LGG Brain Lower Grade Glioma, GBM Glioblastoma multiforme, BRCA Breast invasive carcinoma, CESC Cervical squamous cell carcinoma and endocervical adenocarcinoma, COAD Colon adenocarcinoma, ESCA Esophageal carcinoma, KIRP Kidney renal papillary cell carcinoma, KIRC Kidney renal clear cell carcinoma, KICH Kidney Chromophobe, LIHC Liver hepatocellular carcinoma, LUAD Lung adenocarcinoma, LUSC Lung squamous cell carcinoma, PCPG Pheochromocytoma and Paraganglioma, OV Ovarian serous cystadenocarcinoma, PAAD Pancreatic adenocarcinoma, PRAD Prostate adenocarcinoma, SKCM Skin Cutaneous Melanoma, STAD Stomach adenocarcinoma, TGCT Testicular Germ Cell Tumors, THCA Thyroid carcinoma, UCS Uterine Carcinosarcoma, UCEC Uterine Corpus Endometrial Carcinoma, CHOL Cholangiocarcinoma, SARC Sarcoma, DLBC Lymphoid Neoplasm Diffuse Large B-cell Lymphoma, HNSC Head and Neck squamous cell carcinoma, MESO Mesothelioma, READ Rectum adenocarcinoma, THYM Thymoma, UVM Uveal Melanoma). **(C)** Prediction of CpG islands in the region near the LINC01419 transcription start site by MethPrimer (http://www.urogene.org/methprimer). **(D)** The enrichment of YY1 in the promoter of LINC01419 was evaluated by YY1-ChIPseq (GSM2564089 and GSM2564090 in GSE97411, referred by PMID 29432129) in Huh7 cells. **(E)** Immunostaining of YY1 in Huh7 cells cultured in low-glucose (5 mM) and high-glucose (25 mM) medium (left), statistical analysis of the fluorescence intensity (middle) and the fluorescence intensity ratio of YY1 distribution in the nucleus and cytoplasm (right). Scale bar, 50 μm. The data are presented as the means ± SDs. *P < 0.05, ***P < 0.001, ****P < 0.0001.

**Supplementary Fig. S11**


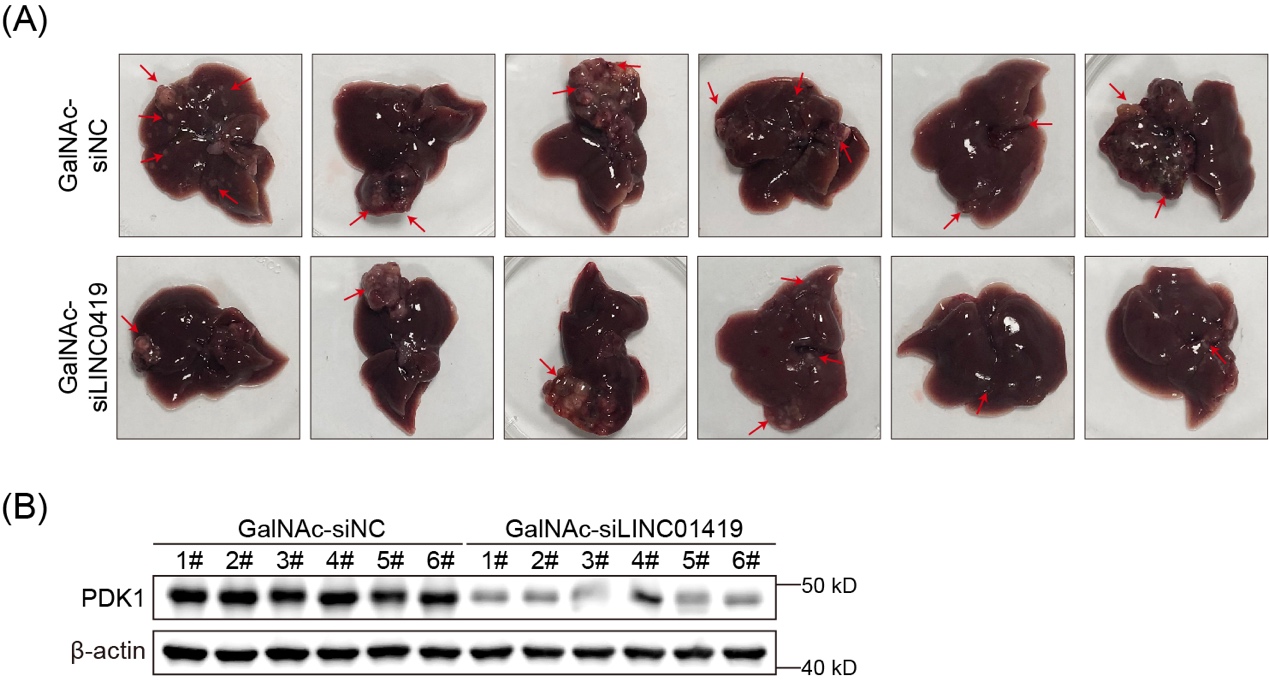


**Figure S11 Administration of GalNAc-siLINC01419 effectively suppresses the growth of orthotopic xenografts derived from HCC cells in vivo.**

**(A)** Image of livers from orthotopic xenograft model mice treated with GalNAc-siNC and GalNAc-siLINC01419. The red arrows in the image indicate the potential tumor tissues on the surface of the mouse liver. **(B)** Western blot analysis of the expression of PDK1 in the GalNAc-siNC- and GalNAc-siLINC01419-treated groups (n=6).
